# Enhancing the high-spin reactivity in C–H bond activation by Iron (IV)-Oxo species: insights from paclitaxel hydroxylation by CYP2C8

**DOI:** 10.3389/fchem.2024.1471741

**Published:** 2024-09-05

**Authors:** Dongxiao Yue, Hajime Hirao

**Affiliations:** Warshel Institute for Computational Biology, School of Medicine, The Chinese University of Hong Kong, Shenzhen, China

**Keywords:** cytochrome P450, high-spin reactivity, C-H bond activation, QM/MM, CYP2C8, paclitaxel

## Abstract

Previous theoretical studies have revealed that high-spin states possess flatter potential energy surfaces than low-spin states in reactions involving iron(IV)-oxo species of cytochrome P450 enzymes (P450s), nonheme enzymes, or biomimetic complexes. Therefore, actively utilizing high-spin states to enhance challenging chemical transformations, such as C–H bond activation, represents an intriguing research avenue. However, the inherent instability of high-spin states relative to low-spin states in pre-reaction complexes often hinders their accessibility around the transition state, especially in heme systems with strong ligand fields. Counterintuitively, our investigation of the metabolic hydroxylation of paclitaxel by human CYP2C8 using a hybrid quantum mechanics and molecular mechanics (QM/MM) approach showed that the high-spin sextet state exhibits unusually high stability, when the reaction follows a secondary reaction pathway leading to 6β-hydroxypaclitaxel. We thoroughly analyzed the factors contributing to the enhanced stabilization of the high-spin state, and the knowledge obtained could be instrumental in designing competent biomimetic catalysts and biocatalysts for C–H bond activation.

## 1 Introduction

The efficient activation of inert C–H bonds is a paramount goal with far-reaching implications across chemical and materials science (Gandeepan et al., 2019; Yamaguchi et al., 2012; Bergman, 2007; Balcells et al., 2010; Anastas and Eghbali, 2010). This capability directly translates into enhanced molecular diversity and streamlined synthesis of complex molecules, such as in drug discovery processes, thereby promoting more sustainable practices. Cytochrome P450 enzymes (P450s) constitute a superfamily of heme-containing proteins and stand out as a remarkable class of biological systems adept at catalyzing such reactions (Ortiz de Montellano, 2015; Werck-Reichhart and Feyereisen, 2000). P450s play a central role in metabolizing a broad range of endogenous or exogenous substrates across various organisms through activating C–H bonds and facilitating other types of reactions. Their ability to activate C–H bonds also makes them invaluable platforms for engineered biocatalyst development (Kumar, 2010; Li et al., 2020; Hu et al., 2023). A precise understanding of the catalytic machinery in P450s could significantly aid in the rational design of biomimetic catalysts.

**SCHEME 1:**
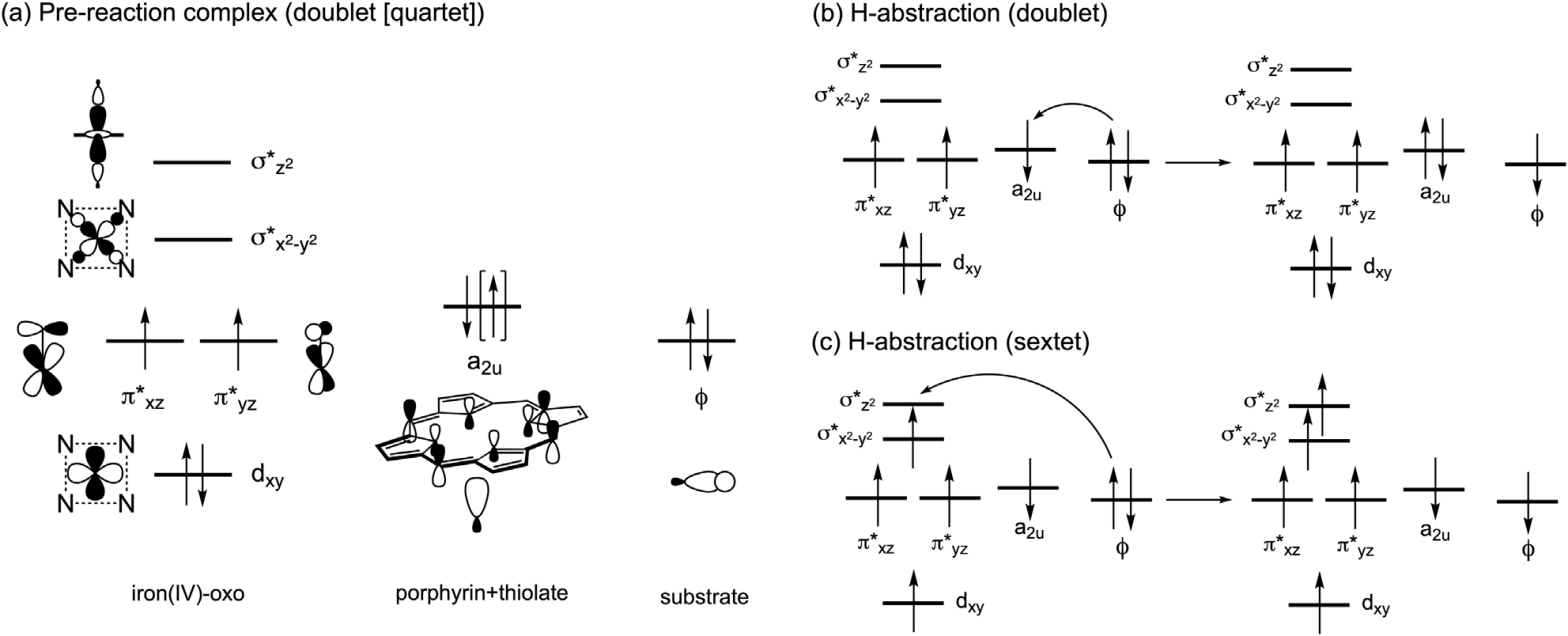
**(A)** Electron configurations in the low-lying doublet and quartet spin states. **(B)** Typical electron shift pattern during H-abstraction in the doublet state. **(C)** Typical electron shift pattern in the sextet state.

The ability of P450s to activate C–H bonds originates from the formation of a high-valent iron(IV)-oxo porphyrin π-cation radical intermediate, known as Compound I (Cpd I). This intermediate is formed through the catalytic cycle that requires the supply of two electrons, two protons, and one O_2_ molecule (Denisov et al., 2005; Sono et al., 1996; Rittle and Green, 2010). Inspired by the remarkable reactivity of Cpd I, biomimetic iron(IV)-oxo complexes have been synthesized (Que, 2007; Nam, 2007). Additionally, our understanding of the electronic structure of Cpd I has advanced through computational studies. In particular, density functional theory (DFT) and hybrid quantum mechanics and molecular mechanics (QM/MM) calculations have highlighted the active involvement of triradicaloid doublet and quartet states in substrate reactions of P450 Cpd I (Shaik et al., 2005; Shaik et al., 2007; Shaik et al., 2010; de Visser et al., 2001). In these states, the π*_xz_, π*_yz_, and a_2u_ orbitals are singly occupied (Scheme 1A), and one of these orbitals, typically the a_2u_ orbital, receives one electron from the substrate during the initial H-abstraction step, yielding a substrate radical (Scheme 1B). While the sextet state at the pre-reaction complex stage containing Cpd I is less stable than the doublet and quartet states, it displays a relatively flat potential energy surface for H-abstraction. This is attributed to additional stabilization from exchange enhancement resulting from the electron shift from the substrate toward the σ*_z_ 2 orbital (Scheme 1C) (Hirao et al., 2005).

Consequently, a common observation in computationally derived energy profiles for P450-catalyzed reactions, regardless of the reaction type, is a reduced energy gap between the sextet and lower-spin states at the bond-activation transition state (TS), compared to the pre-reaction state. For instance, a DFT study on C–H hydroxylation and olefin epoxidation reactions of P450 Cpd I demonstrated that the relative stability of the sextet TS increases as the reactions progress (Hirao et al., 2005). In our recent QM/MM study on the CYP3A4-catalyzed aromatic hydroxylation reaction of paclitaxel (PTX, or Taxol), the energy gap was initially 6.4 kcal/mol. However, at the TS for C–O bond formation between an aromatic ring and Cpd I, the gap significantly narrowed to just 0.3 kcal/mol, with the sextet state exhibiting slightly higher stability than the doublet state (Yue and Hirao, 2023).

Given the relatively high energy of P450 Cpd I or its pre-reaction complexes in high-spin states, stabilizing them is considered a promising strategy for enhancing the stability of the subsequent TSs. This can be achieved by substituting the heme ligand with a nonheme ligand to reduce the iron(IV)-oxo ligand field strength (Kumar et al., 2005; Hirao et al., 2006; Sastri et al., 2007; Hirao et al., 2008a; Hirao et al., 2015). Nonheme iron enzymes, such as taurine dioxygenase (TauD), are known to produce iron(IV)-oxo species with a high-spin (*S* = 2) ground state (Krebs et al., 2007). Furthermore, various high-spin iron(IV)-oxo complexes have been successfully synthesized through meticulous nonheme ligand design (Puri and Que, 2015; England et al., 2009; England et al., 2010; England et al., 2011; Lacy et al., 2010; Bigi et al., 2012; Bae et al., 2016; Hou et al., 2023). Theoretical studies have also suggested that external electric fields could further stabilize high-spin TSs in the reactions of nonheme iron (IV)-oxo complexes (Hirao et al., 2008b).

**SCHEME 2:**
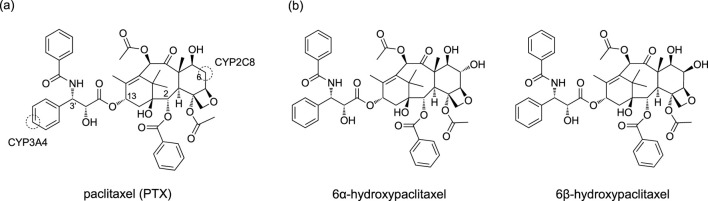
**(A)** Chemical structure of PTX and its metabolic sites targeted by CYP2C8 and CYP3A4. **(B)** 6α-hydroxypaclitaxel and 6β-hydroxypaclitaxel.

Thus, nonheme ligands clearly offer better support for stabilizing high-spin states of the pre-reaction complex. Nevertheless, this study explores a less examined area by investigating the high-spin reactivity of P450 Cpd I. Specifically, we investigate the CYP2C8-catalyzed hydroxylation reaction of PTX, a renowned anticancer compound (Stage et al., 2018; Singla et al., 2002; Weaver, 2014). Experimental evidence suggests that PTX undergoes hepatic metabolic transformations catalyzed by CYP2C8 and CYP3A4 following administration (Harris et al., 1994b; Rahman et al., 1994). As illustrated in Scheme 2A, these enzymes target different sites, resulting in distinct products. Our recent study focused on the CYP3A4-catalyzed aromatic hydroxylation of PTX at the 3′-phenyl ring (Yue and Hirao, 2023). We have also recently investigated the mechanism of the CYP2C8-catalyzed hydroxylation of PTX into 6α-hydroxypaclitaxel using QM/MM calculations (Yue and Hirao, submitted). While further exploring CYP2C8-catalyzed hydroxylation of PTX, we unexpectedly discovered that the high-spin sextet state could exhibit remarkably enhanced stability compared to other spin states at the H-abstraction TS when PTX is converted to 6β-hydroxypaclitaxel (Scheme 2B). Although 6β-hydroxypaclitaxel formation has not been experimentally observed, our computational findings reveal an intriguing aspect of high-spin iron (IV)-oxo reactivity. The unexpected stability of the high-spin state in this reaction prompted us to investigate the contributing factors beyond pre-reaction complex stabilization, which could aid in the rational design of high-spin catalysts.

## 2 Computational methods

### 2.1 Molecular docking and molecular dynamics simulations

To identify a plausible binding structure of PTX in CYP2C8, we initially performed molecular docking simulations. We utilized MOE 2020 software (Molecular Operating Environment (MOE), 2022) and docked PTX into several crystal structures of CYP2C8 (PDB codes 2NNH, 2NNI, 2VN0, and 2NNJ). Unlike CYP3A4, these crystal structures exhibit minimal structural variations, leading to comparable docking results. Consequently, we proceeded with the structure from 2NNJ. To further refine the CYP2C8–PTX complex structure, we performed molecular dynamics (MD) simulations using AMBER (Case et al., 2020). The MD simulations essentially followed the same protocol employed in our prior study of CYP3A4 (Yue and Hirao, 2023), including clustering to select a representative structure.

### 2.2 QM/MM calculations

To investigate the reaction mechanism, we employed the ONIOM(QM:MM) method, a subtractive QM/MM scheme (Chung et al., 2012; Chung et al., 2015). Gaussian 16 software was used for QM/MM computations (Frisch et al., 2016). The CYP2C8–PTX complex geometry, derived from MD simulations, served as the starting point. To simplify the system, a significant portion of the solvent water molecules outside the enzyme was removed. The QM region encompassed the porphine and iron-oxo units, the C_β_H_3_S^−^segment of the cysteine axial ligand, and the PTX molecule. DFT methods were applied to describe the QM atoms. Geometry optimization and vibrational frequency calculations were performed using the ONIOM mechanical-embedding scheme and the B3LYP/6-31G(d) QM method. The ONIOM energy obtained with this basis set is referred to as *E*1. For single-point energy calculations, the B3LYP/def2-TZVP method and the electronic-embedding scheme were utilized (Becke, 1993; Lee et al., 1988; Vosko et al., 1980; Hehre et al., 1972; Weigend and Ahlrichs, 2005). MM calculations within the ONIOM framework employed AMBER and TIP3P parameters (Cornell et al., 1995; Jorgensen et al., 1983). Force field parameters for PTX and the heme group were consistent with those in the MD simulations. Free energies were determined by summing the ONIOM single-point energy with a larger basis set (*E*2), the DFT-D3BJ dispersion correction to the QM energy (*E*_disp_) (Grimme et al., 2011; Becke and Johnson, 2005), and the free energy correction obtained from ONIOM vibrational frequency analysis (*G*_corr_). The sum of these energy values (*G*) was subsequently used to construct reaction energy diagrams.

## 3 Results and discussion

### 3.1 Active-site feature and possible binding pose

**FIGURE 1:**
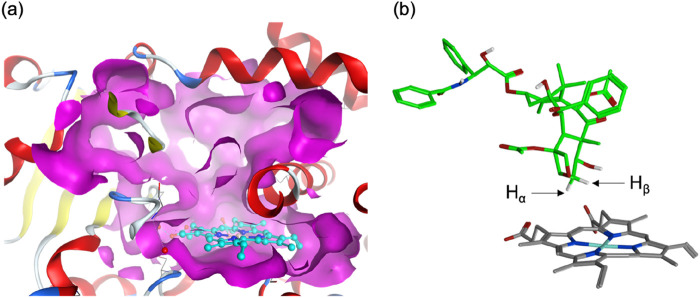
**(A)** Surface representation of the active site of human CYP2C8 (PDB code 2NNJ). **(B)** A top-ranked binding structure obtained from docking simulations.

Human P450s generally possess spacious active sites, enabling them to accommodate a wide range of substrates. CYP2C8 (PDB code 2NNJ), with a 746 Å^3^ active site, readily binds PTX (Figure 1A). However, an α-helix overlying the heme restricts ligand access to this region. This structural feature is also observed in CYP2C9, which shares 78% sequence identity with CYP2C8 (Williams et al., 2003). As a result, molecular docking identified a binding mode (Pose A) avoiding the α-helix region and positioning the metabolic site’s H_β_ closer to the heme iron compared to H_α_ (Figure 1B). At the C6 position of PTX in Scheme 2, H_α_ and H_β_ are potential sites for hydroxylation. While C–H_α_ hydroxylation would produce the experimentally observed 6α-hydroxypaclitaxel metabolite (Harris et al., 1994a; Kumar et al., 1994), C–H_β_ hydroxylation would yield the unobserved 6β-hydroxypaclitaxel. Thus, Pose A is inconsistent with experimental findings. Further analysis revealed that the Pose A conformation of PTX is not very stable in its isolated form, which could lead to an overestimation of its binding affinity in docking simulations. Consequently, a lower-ranked binding mode, involving a substrate with an intrinsically more stable conformation, should be a more probable candidate for C–H_α_ hydroxylation (Yue and Hirao, submitted). Nevertheless, in this study, we delved deeper into the reactivity of Pose A, which unexpectedly led us to uncover intriguing insights into high-spin reactivity.

### 3.2 QM/MM mechanistic study of PTX hydroxylation

**FIGURE 2:**
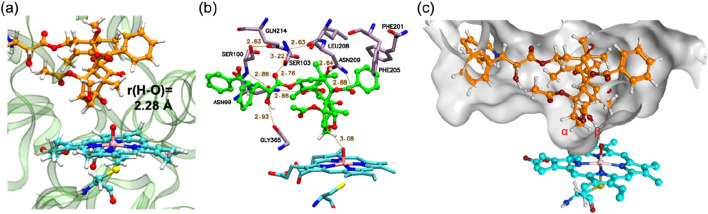
**(A)** QM/MM-optimized structure of ^2^ **1**. **(B)** 3D ligand interaction diagram of ^2^ **1**. **(C)** A surface representation of the active site in ^2^ **1**. Key distances are presented in Å.

Following MD refinement of the Pose A structure, we conducted QM/MM mechanistic studies. P450-catalyzed alkane hydroxylation typically initiates with H-abstraction from a C–H bond (Schöneboom et al., 2004). As illustrated in Figure 2A, the geometry-optimized pre-reaction complex between Cpd I and PTX (**1**) in the doublet ground state (^2^ **1**) exhibits an O–H distance of 2.28 Å between the H_β_ atom and the Cpd I oxo group. This proximity suggests a favorable conformation for subsequent H-abstraction. Figure 2B highlights key interactions between PTX and surrounding amino-acid residues. PTX is securely anchored within the CYP2C8 active site via non-bonded interactions including hydrogen bonds and π-π interactions. Specifically, Asn99, Ser103, Asn209, and Gly365 form hydrogen bonds with PTX’s polar groups. Moreover, a hydrogen-bond network involving Asn99, Ser100, Gln214, Ser103, Leu208, and Asn209 stabilizes the Cpd I–PTX complex. Phe201 and Phe205 engage in hydrophobic interactions with the phenyl moiety of the benzoyloxy group in PTX. Figure 2C depicts the available space around PTX using a surface model, clearly demonstrating that PTX’s excellent fit within the active site.

Through detailed QM/MM calculations on 6β-hydroxypaclitaxel formation pathways, we identified four distinct electron-shift patterns for the H-abstraction step. One doublet-state pathway (Path A) involves an electron shift from the substrate orbital (ϕ) to the a_2u_-type orbital of Cpd I, yielding intermediate ^2^ **3a** with a negative spin density (ρ) value (∼–1.0) on the PTX moiety (Scheme 1B; Supplementary Table S2). Another doublet-state pathway (Path B) features an electron shift from ϕ to the π* orbital of Cpd I, forming intermediate ^2^ **3b** with a positive ρ value (∼1.0) on PTX (Supplementary Table S2). In the quartet spin state, an electron migrates from the substrate to the a_2u_-type orbital of Cpd I, producing intermediate ^4^ **3** with a positive ρ value (∼1.0) on PTX (Supplementary Table S2). Finally, the sextet spin state involves electron migration from ϕ to the σ*_z_ 2 orbital of Cpd I, resulting in intermediate ^6^ **3** with a negative ρ value (∼–1.0) on PTX (Supplementary Table S2).

**FIGURE 3:**
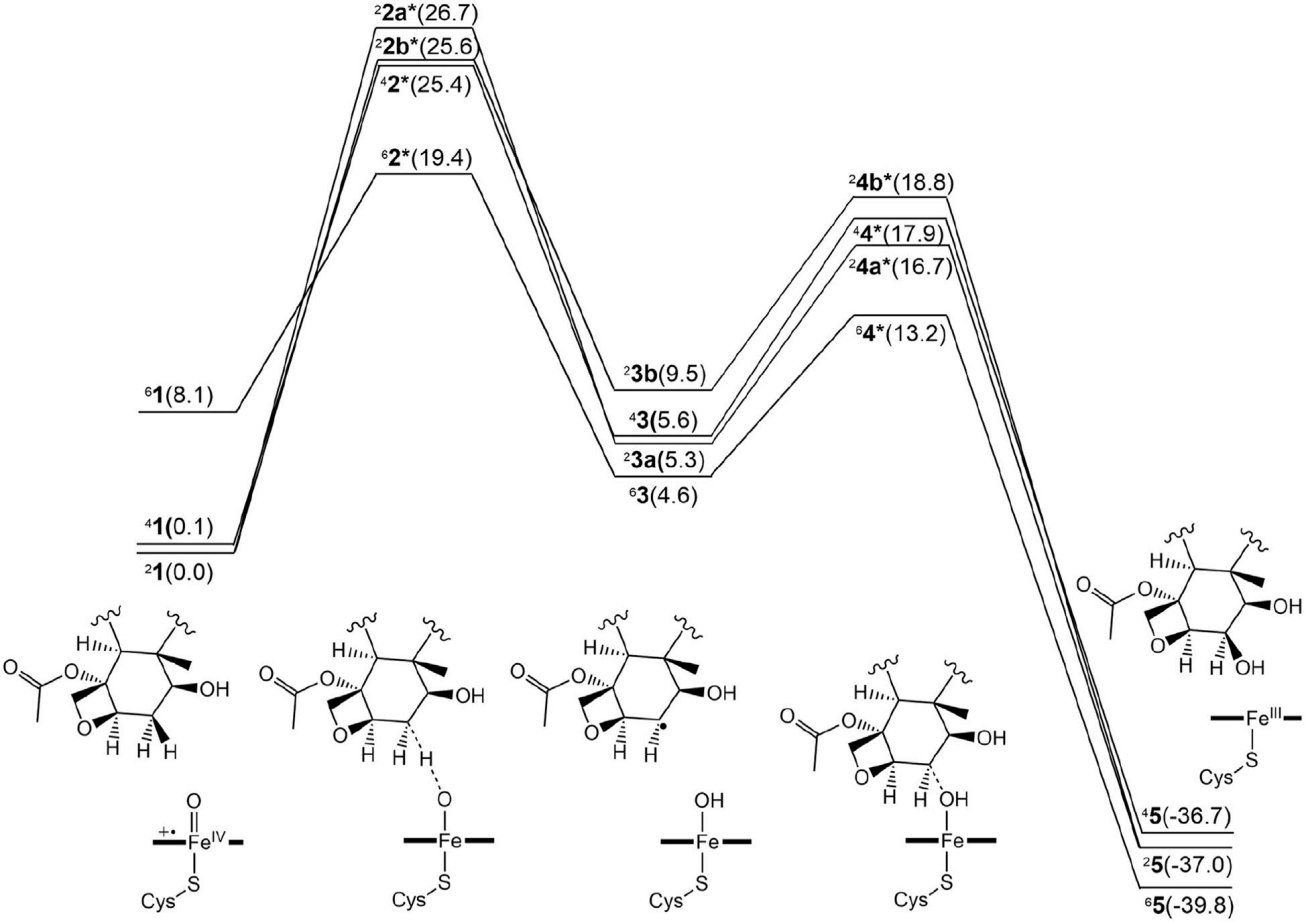
Free energy profile for the β-hydroxylated PTX metabolite formation in the doublet, quartet, and sextet spin states. The numerical values within parentheses indicate relative free energy values in kcal/mol.

Figure 3 presents the free energy profile for PTX hydroxylation. As described, two electron-shift patterns can occur during the doublet-state H-abstraction step, resulting in two TSs (^2^ 2a* and ^2^ 2b*) for Paths A and B, respectively. TSs were also obtained in the quartet (^4^ 2*) and sextet (^6^ 2*) states. Comparing relative energies reveals ^2^ 2b* as slightly more stable (25.6 kcal/mol) than ^2^ 2a* (26.7 kcal/mol). Without corrections, the uncorrected *E*1 values produce barriers exceeding 30 kcal/mol for both doublet states (Supplementary Table S1), significantly surpassing typically observed values of around 20 kcal/mol (Schöneboom et al., 2004). These results suggest that the substrate experiences significant strain around the TS in the protein environment. Intermediates ^2^ **3a** and ^2^ **3b** exhibit reversed stability (5.3 and 9.5 kal/mol, respectively). The stability of the quartet-state TS (^4^ 2*, 25.4 kcal/mol) is comparable to that of ^2^ 2b*, with subsequent intermediate ^4^ **3** at 5.6 kcal/mol.

Surprisingly, the sextet-state TS (^6^ 2*, 19.4 kcal/mol) is significantly more stable than the others, with energy differences of at least 6 kcal/mol. This exceptional stability arises from a remarkably low barrier of 11.3 kcal/mol on the sextet-state free energy surface, contrasting sharply with the higher barriers observed for the other spin states. While the sextet state typically benefits from transition-state stabilization due to exchange enhancement (Hirao et al., 2005), the substantially lower energy of the sextet TS in the current system is an unusual observation. Normally, the sextet state’s stability at the TS is comparable to or slightly inferior to that of lower spin states. In contrast, the CYP3A4-catalyzed aromatic hydroxylation reaction exhibited a slight energetic preference for the sextet TS, with a small energy gap of 0.3 kcal/mol (Yue and Hirao, 2023). The significantly greater stability of the sextet TS in the current CYP2C8-catalyzed reaction is expected to increase the likelihood of this reaction channel being accessed.

**FIGURE 4:**
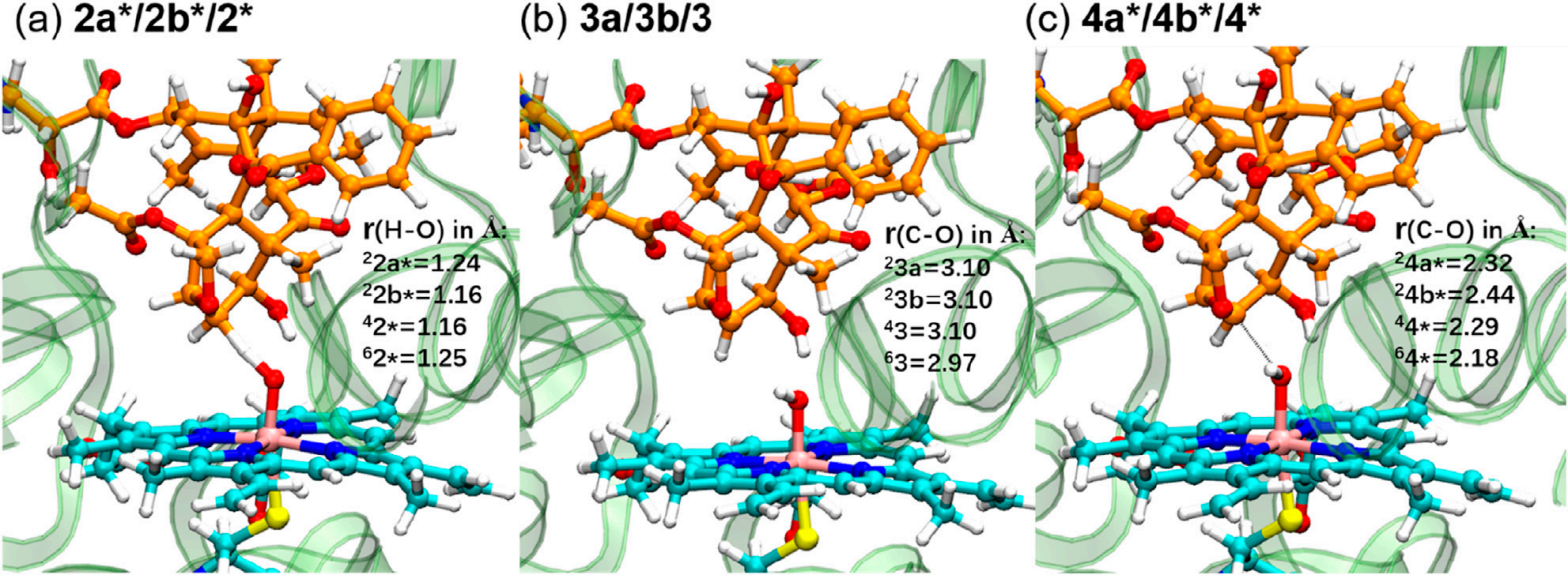
QM/MM-optimized geometries of key TSs (**(A)** and **(C)**) and intermediates **(B)**.

Figure 4 displays the optimized geometries of key TSs and intermediates. The larger O–H distance observed in ^6^ 2* (1.25 Å) than those in the other corresponding species (1.16–1.24 Å) indicates an earlier TS in the sextet pathway, consistent with the lower energy barrier observed in the sextet-state energy profile (Figure 3). A closer examination of Fe–O–H angles across different spin states reveals a larger angle for ^6^ 2* (135.1°) compared to the others (around 127.0°). This larger Fe–O–H angle in the sextet state is characteristic of the high-spin H-abstraction mechanism, involving an electron shift from the substrate’s ϕ orbital to the axial σ*_z_ 2 orbital of iron(IV)-oxo. Optimal orbital overlap is achieved when the substrate approaches the iron (IV)-oxo unit from above, leading to a larger Fe–O–H angle (Hirao et al., 2005).

We further investigated the rebound step leading to cycloalkanol product formation (Figures 3, 4) (Groves, 1985). Starting from intermediates ^2^ **3a** and ^3^ **3b** in the doublet spin state, we obtained product ^2^ **5** (−37.0 kcal/mol) via TSs ^2^ 4a* (16.7 kcal/mol) and ^2^ 4b* (18.8 kcal/mol), respectively. While Path A exhibits a lower overall energy, its rebound barrier of 11.3 kcal/mol is unusually high for a P450 reaction in the doublet state compared to other reported values, likely due to steric constraints within the enzyme’s active site. The quartet TS ^4^ 4* (17.9 kcal/mol) lies energetically between ^2^ 4a* and ^2^ 4b*, leading to product ^4^ **5** with a relative free energy of −36.7 kcal/mol, which is less stable than the doublet product (^2^ **5**, −37.0 kcal/mol). From the most stable intermediate ^6^ **3** (4.6 kcal/mol), we obtained the most stable product ^6^ **5** (−39.8 kcal/mol) via TS ^6^ 4* (13.2 kcal/mol). Surprisingly, the sextet state exhibits the lowest rebound barrier in the sextet state (8.6 kcal/mol), contrary to the typical trend of higher rebound barriers in the sextet state.

### 3.3 Energy decomposition analysis

Our QM/MM mechanistic study on CYP2C8-catalyzed PTX hydroxylation unexpectedly revealed a dominant role for the high-spin sextet state from an early stage of the reaction. To understand the origins of this pronounced sextet state stability, we conducted further theoretical analyses. The ONIOM-based *G* value can be expressed as follows (Equation 1):

$$G=E_{\text{QM}}+E_{\text{disp}}+E_{\text{MM}}+E_{\text{pol}}+G_{\text{corr}}$$ (1)

where *E*_QM_ is the gas-phase energy of the QM atoms at the QM/MM geometry, and *E*_MM_ and *E*_pol_ are defined by Equations 2, 3, respectively:

$$E_{\text{MM}}=E_{\text{MM},\text{real}}\;–\;E_{\text{MM},\text{model}}$$ (2)

$$E_{\text{pol}}=E2\left(\text{EE}\right)\;–\;E2\left(\text{ME}\right)$$ (3)

*E*2(EE) and *E*2(ME) are ONIOM-EE and ONIOM-ME energies obtained from single-point energy calculations with the def2-TZVP basis set (Hirao, 2011a; Hirao, 2011b). The relative free energy (Δ*G*) of the H-abstraction TS with respect to ^2^ **1** is given by Equation 4:

$$\Delta G=\Delta E_{\text{QM}}+\Delta E_{\text{disp}}+\Delta E_{\text{MM}}+\Delta E_{\text{pol}}+\Delta G_{\text{corr}}$$ (4)

**FIGURE 5:**
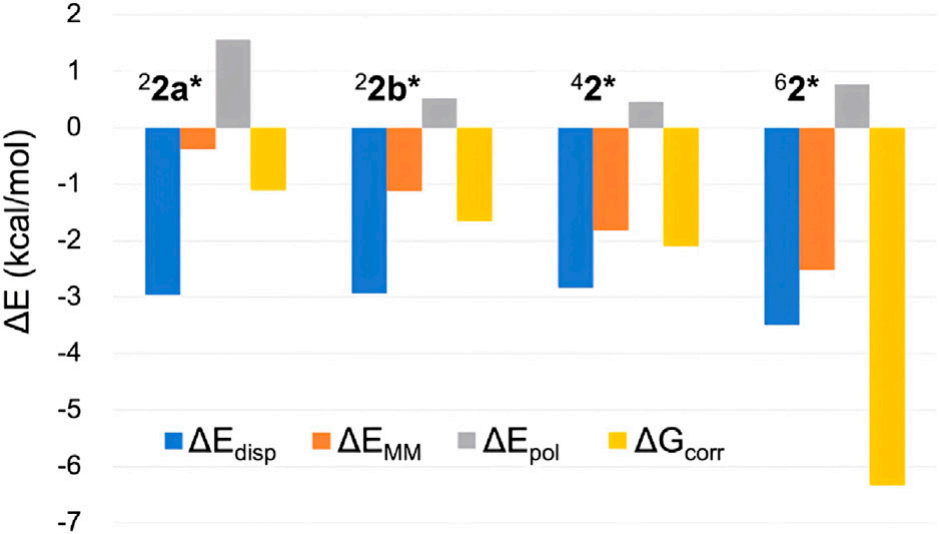
Comparison of several energy terms constituting Δ*G* (in kcal/mol) of H-abstraction TSs in different spin states.

While ∆*E*_QM_ can be obtained from DFT calculations on the QM atoms, the remaining four terms also contribute to the overall Δ*G* value. Figure 5 displays the latter four terms for the H-abstraction TSs in different spin states. The consistently negative ∆*E*_disp_ values indicate stronger dispersion stabilization in TSs compared to ^2^ **1**, with the largest stabilization observed for the sextet state. Therefore, although modest, dispersion contributes to the sextet state’s stability. The relatively large ∆*E*_disp_ for the sextet TS is attributed to appropriate positioning of the QM atoms, which enhances interatomic dispersion stabilization. The MM energy term (∆*E*_MM_) is also consistently negative, with the largest stabilization for ^6^ 2*. Therefore, the MM effect partly contributes to the pronounced stability of ^6^ 2*. While the polarization effect (∆*E*_pol_) does not enhance the sextet TS’s stability, the ∆*G*_corr_ term stabilizes all TSs, with the greatest stabilization observed for the sextet state. This likely results from the sextet TS’s flexible and entropically favorable structure. Among the four terms examined, the free energy correction term (∆*G*_corr_) provides the most significant stabilization for ^6^ 2*.

**FIGURE 6:**
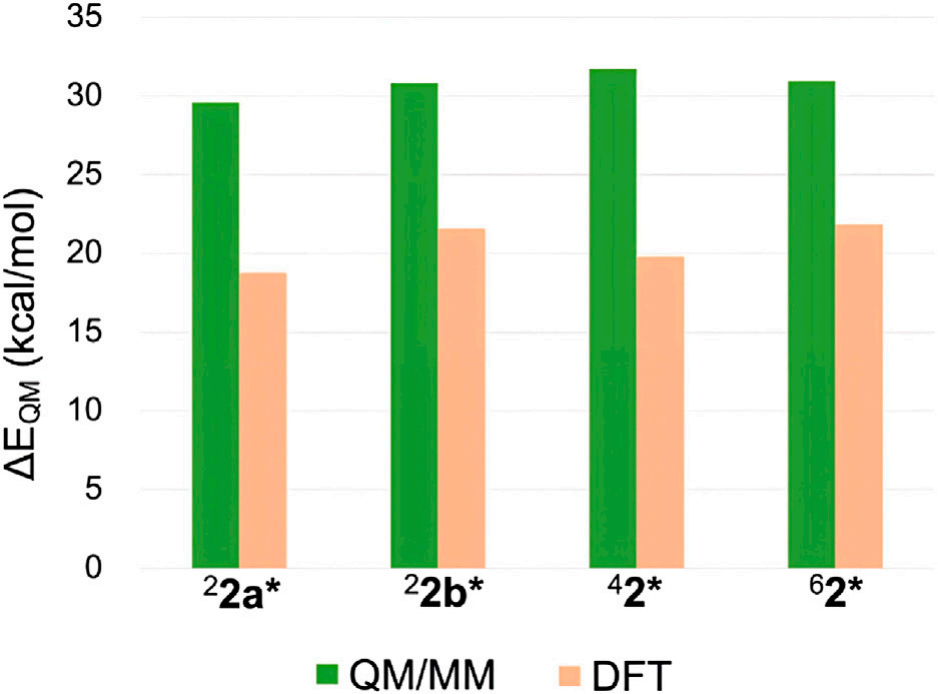
Δ*E*_QM_ values obtained by QM/MM and DFT calculations.

Despite the insights gained from the above analysis, the substantial relative stability of ^6^ 2* remained only partially explained. Therefore, we further investigated the Δ*E*_QM_ values across different spin states. In addition to the QM/MM approach, DFT calculations were performed using a P450 Cpd I model and cyclohexane to represent the reaction. Figure 6 compares the Δ*E*_QM_ values for different spin states obtained from both methods. Interestingly, significant discrepancies were observed between the QM/MM and DFT results. QM/MM calculations consistently yielded higher Δ*E*_QM_ by 9.1–11.9 kcal/mol compared to DFT. In other words, the TS geometries are highly strained in the protein environment. However, the smallest destabilization was observed for the sextet state (9.1 kcal/mol). Thus, the protein environment destabilizes TSs more in the other spin states, contributing to the enhanced relative stability of ^6^ 2*. The smaller destabilization in the sextet state is likely due to the protein-imposed geometric constraints that orient the substrate more favorably for the sextet-state TS geometry.

**SCHEME 3:**
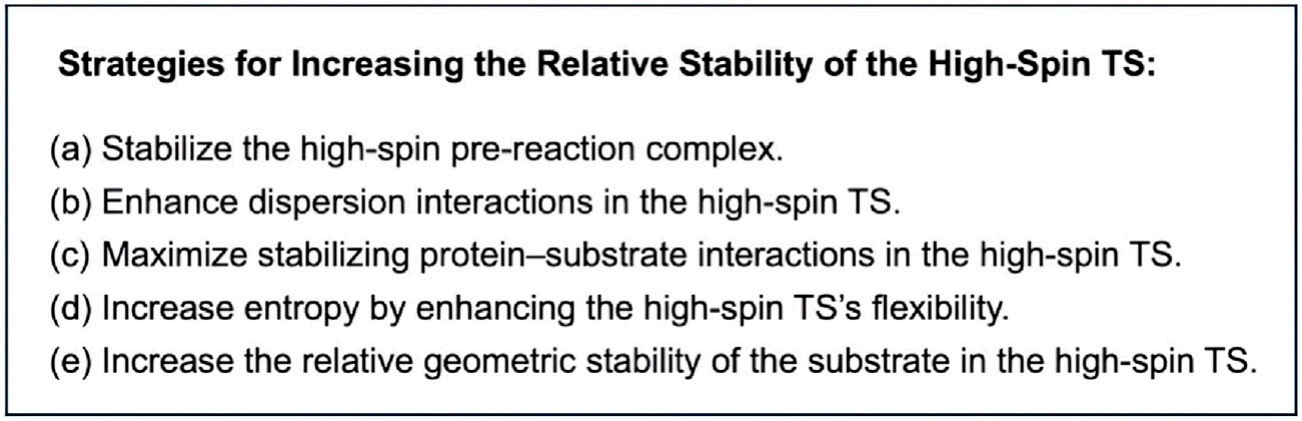
Possible strategies for increasing the relative stability of high-spin TSs in reactions of iron(IV)-oxo species.

Based on the present theoretical analysis and previous studies, several possible strategies for promoting high-spin reactivity of iron(IV)-oxo complexes can be summarized (Scheme 3). The most fundamental approach involves stabilizing the high-spin pre-reaction complex, which lowers the energy of the entire high-spin energy surface (Scheme 3A). This can be achieved through the use of nonheme ligands that reduce ligand field strength. This study highlights additional factors beyond this approach (Scheme 3B-E), with entropy and QM effects being particularly influential (Scheme 3D,E). We propose that these effects are key to enhancing high-spin reactivity. While pronounced entropy benefits for high-spin TSs have been observed in previous studies (Yue and Hirao, 2023), further enhancing the high-spin reactivity requires increasing the quantum mechanical stability of the high-spin TS relative to others. The surrounding environment plays a crucial role in influencing this effect, as the QM effect is largely related to differing degrees of geometric strain of the substrate. If the environment imposes steric constraints on the substrate, enforcing a linear approach toward the iron(IV)-oxo unit from above, a lower degree of destabilization in the high-spin TS compared to others can be expected, as the high-spin state can undergo an efficient electron shift in an exchange enhanced fashion within this configuration. Therefore, even in nonenzymatic environments, ligand design should consider such steric effects.

## 4 Conclusion

Actively harnessing the high-spin reactivity of iron(IV)-oxo species presents a promising strategy for activating inert C–H bonds of organic substrates. Traditionally, nonheme ligands have been employed to reduce ligand field strength and stabilize the pre-reaction complex, while high-spin reactivity of P450s has not been widely explored. Our current QM/MM study on CYP2C8-catalyzed PTX hydroxylation revealed that the high-spin TS can be remarkably stabilized even within a heme ligand environment, particularly when forming 6β-hydroxypaclitaxel. Detailed energy decomposition analysis identified the critical roles of entropy and the substrate’s quantum mechanical (strain) effects in stabilizing the high-spin TS. To enhance the latter effect, it is essential to impose steric constraints on the substrate using surrounding atoms, thereby reducing the relative destabilization of the high-spin state. These insights could be strategically applied to the rational design of high-spin iron(IV)-oxo catalysts for C–H bond activation.

## Data Availability

The original contributions presented in the study are included in the article/Supplementary Material, further inquiries can be directed to the corresponding author.
