# Supplementary material for: Enhancing the high-spin reactivity in C–H bond activation by Iron (IV)-Oxo species: insights from paclitaxel hydroxylation by CYP2C8
Source: Front Chem. 2024 Sep 5;12:1471741. doi: 10.3389/fchem.2024.1471741 (PMC11427847; doi:10.3389/fchem.2024.1471741)
Supplement: Supplementary file 1 [file DataSheet1.PDF]

Supplementary Material

Enhancing the High-Spin Reactivity in C–H Bond Activation by Iron(IV)-Oxo Species:

Insights from Paclitaxel Hydroxylation by CYP2C8

Dongxiao Yue[1] and Hajime Hirao*,[1]

[1] *Warshel Institute for Computational Biology, School of Medicine, The Chinese University of Hong Kong, Shenzhen, Guangdong, 518172, P. R. China.*

*hirao@cuhk.edu.cn; Tel.: +86-0755-23519030

Contents

1. ONIOM calculation results
2. Group spin density data
3. Group charge data
4. XYZ coordinates of QM atoms

1. ONIOM calculation results

Table S1. ONIOM energy data for all species.

(a) Doublet state

	Imaginary frequency [cm^{-1}]	E1 [hartrees]	G_{corr} [hartrees]	E2 [hartrees]	E_{disp} [hartrees]	$\Delta(E2+E_{disp}+G_{corr})$ [kcal/mol]
**1**		-5724.138600	62.927090	-5725.786365	-0.439424	0.0
**2a***	1674.7887i	-5724.090176	62.925331	-5725.737322	-0.444146	26.7
**2b***	2014.3392i	-5724.086970	62.924457	-5725.738198	-0.444100	25.6
**3a**		-5724.122360	62.925247	-5725.773556	-0.441868	5.3
**3b**		-5724.113231	62.926049	-5725.767850	-0.441700	9.5
**4a***	476.7543i	-5724.109772	62.931796	-5725.757272	-0.446683	16.7
**4b***	321.4939i	-5724.101414	62.929488	-5725.754289	-0.444012	18.8
**5**		-5724.192072	62.929966	-5725.843865	-0.443687	-37.0

(b) Quartet state

	Imaginary frequency [cm^{-1}]	E1 [hartrees]	G_{corr} [hartrees]	E2 [hartrees]	E_{disp} [hartrees]	$\Delta(E2+E_{disp}+G_{corr})$ [kcal/mol]
**1**		-5724.137811	62.926421	-5725.785722	-0.439267	0.1
**2***	1934.4654i	-5724.086416	62.923749	-5725.737975	-0.443945	25.4
**3**		-5724.122298	62.925556	-5725.773643	-0.441753	5.6
**4***	475.5538i	-5724.107058	62.930155	-5725.756097	-0.444222	17.9
**5**		-5724.195110	62.929745	-5725.845464	-0.441494	-36.7

(c) Sextet state

	Imaginary frequency [cm^{-1}]	E1 [hartrees]	G_{corr} [hartrees]	E2 [hartrees]	E_{disp} [hartrees]	$\Delta(E2+E_{disp}+G_{corr})$ [kcal/mol]
**1**		-5724.122516	62.922312	-5725.769117	-0.439034	8.1
**2***	1508.2481i	-5724.091506	62.916998	-5725.739839	-0.444992	19.4
**3**		-5724.116023	62.920238	-5725.766999	-0.444559	4.6
**4***	452.0388i	-5724.104958	62.924159	-5725.754253	-0.447593	13.2
**5**		-5724.197039	62.925887	-5725.849028	-0.438984	-39.8

2. Group spin density data

Table S2. Mulliken group spin density data obtained for the QM atoms using the ONIOM-ME(B3LYP/6-31G*/MM) and ONIOM-EE(B3LYP/def2-TZVP:MM) methods.

(a) Doublet state

	Fe	O	Porphine	SCH3	PTX	Total
**1** (ME)	1.39	0.70	-0.34	-0.76	0.01	1.00
**1** (EE)	1.36	0.73	-0.39	-0.71	0.01	1.00
**2a***(ME)	2.05	-0.18	-0.23	-0.23	-0.42	1.00
**2a***(EE)	2.07	-0.08	-0.34	-0.28	-0.36	1.00
**2b***(ME)	1.13	0.45	-0.38	-0.69	0.49	1.00
**2b***(EE)	1.18	0.45	-0.45	-0.64	0.46	1.00
**3a**(ME)	1.88	0.13	-0.14	0.10	-0.98	1.00
**3a**(EE)	1.91	0.12	-0.15	0.09	-0.98	1.00
**3b**(ME)	1.00	0.07	-0.36	-0.69	0.98	1.00
**3b**(EE)	1.02	0.07	-0.44	-0.64	0.98	1.00
**4a***(ME)	1.58	0.19	-0.12	0.00	-0.66	1.00
**4a***(EE)	1.76	0.15	-0.13	-0.01	-0.77	1.00
**4b***(ME)	1.16	-0.09	-0.34	-0.37	0.64	1.00
**4b***(EE)	1.18	-0.05	-0.56	-0.37	0.81	1.00
**5**(ME)	1.09	0.00	-0.09	0.00	0.00	1.00
**5**(EE)	1.09	0.00	-0.11	0.02	0.00	1.00

(b) Quartet state

	Fe	O	Porphine	SCH_3	PTX	Total
**1** (ME)	1.33	0.72	0.21	0.74	0.01	3.00
**1** (EE)	1.29	0.76	0.25	0.69	0.01	3.00
**2*** (ME)	1.10	0.48	0.24	0.67	0.50	3.00
**2*** (EE)	1.13	0.48	0.28	0.63	0.47	3.00
**3** (ME)	1.89	0.14	-0.14	0.12	0.99	3.00
**3** (EE)	1.91	0.13	-0.15	0.11	1.00	3.00
**4*** (ME)	2.33	-0.07	-0.13	0.19	0.67	3.00
**4*** (EE)	2.29	-0.04	-0.15	0.13	0.76	3.00
**5** (ME)	2.58	0.00	-0.02	0.44	0.00	3.00
**5** (EE)	2.61	0.00	-0.05	0.45	0.00	3.00

(c) Sextet state

	Fe	O	Porphine	SCH_3	PTX	Total
**1** (ME)	3.28	0.49	0.55	0.69	0.00	5.00
**1** (EE)	3.21	0.53	0.62	0.63	0.00	5.00
**2*** (ME)	4.10	-0.07	0.49	0.89	-0.41	5.00
**2*** (EE)	4.04	-0.04	0.49	0.89	-0.38	5.00
**3** (ME)	4.24	0.26	0.56	0.90	-0.96	5.00
**3** (EE)	4.21	0.25	0.62	0.89	-0.96	5.00
**4*** (ME)	4.14	0.19	0.45	0.84	-0.62	5.00
**4*** (EE)	4.13	0.18	0.55	0.87	-0.73	5.00
**5** (ME)	4.13	0.00	0.43	0.44	0.00	5.00
**5** (EE)	4.10	0.00	0.45	0.44	0.00	5.00

3. Group charge data

Table S3. Mulliken group charge data obtained for the QM atoms using the ONIOM-ME(B3LYP/6-31G*/MM) and ONIOM-EE(B3LYP/def2-TZVP:MM) methods.

(a) Doublet state

	Fe	O	Porphine	SCH_3	PTX	Total
**1** (ME)	1.24	-0.50	-0.70	-0.02	-0.02	0.00
**1** (EE)	0.08	-0.53	0.59	-0.10	-0.05	0.00
**2a*** (ME)	1.26	-0.67	-0.71	-0.15	0.27	0.00
**2a*** (EE)	-0.10	-0.59	0.75	-0.18	0.11	0.00
**2b*** (ME)	1.22	-0.69	-0.75	-0.02	0.23	0.00
**2b*** (EE)	0.02	-0.59	0.58	-0.10	0.08	0.00
**3a** (ME)	1.24	-0.78	-0.76	-0.10	0.40	0.00
**3a** (EE)	-0.16	-0.63	0.65	-0.13	0.26	0.00
**3b** (ME)	1.20	-0.79	-0.79	0.00	0.38	0.00
**3b** (EE)	-0.03	-0.65	0.53	-0.08	0.24	0.00
**4a*** (ME)	1.20	-0.75	-0.88	-0.15	0.59	0.00
**4a*** (EE)	-0.14	-0.63	0.58	-0.16	0.35	0.00
**4b*** (ME)	1.17	-0.78	-0.84	-0.15	0.60	0.00
**4b*** (EE)	-0.07	-0.70	0.61	-0.17	0.33	0.00
**5** (ME)	1.01	-0.64	-0.91	-0.16	0.69	0.00
**5** (EE)	-0.06	-0.50	0.28	-0.21	0.49	0.00

(b) Quartet state

	Fe	O	Porphine	SCH_3	PTX	Total
**1** (ME)	1.25	-0.50	-0.70	-0.02	-0.02	0.00
**1** (EE)	0.10	-0.52	0.57	-0.10	-0.05	0.00
**2*** (ME)	1.23	-0.68	-0.75	-0.03	0.23	0.00
**2*** (EE)	0.03	-0.58	0.56	-0.10	0.08	0.00
**3** (ME)	1.24	-0.78	-0.76	-0.10	0.40	0.00
**3** (EE)	-0.16	-0.63	0.65	-0.13	0.26	0.00
**4*** (ME)	1.23	-0.77	-0.81	-0.21	0.56	0.00
**4*** (EE)	-0.10	-0.67	0.60	-0.18	0.35	0.00
**5** (ME)	1.11	-0.64	-0.83	-0.32	0.69	0.00
**5** (EE)	0.07	-0.50	0.28	-0.34	0.49	0.00

(c) Sextet state

	Fe	O	Porphine	SCH_3	PTX	Total
**1** (ME)	1.35	-0.51	-0.80	-0.02	-0.02	0.00
**1** (EE)	0.43	-0.55	0.26	-0.09	-0.05	0.00
**2*** (ME)	1.41	-0.70	-0.91	-0.06	0.27	0.00
**2*** (EE)	0.55	-0.68	0.10	-0.11	0.14	0.00
**3** (ME)	1.42	-0.86	-0.93	-0.06	0.43	0.00
**3** (EE)	0.57	-0.78	0.06	-0.11	0.27	0.00
**4*** (ME)	1.36	-0.81	-1.02	-0.11	0.57	0.00
**4*** (EE)	0.51	-0.72	0.00	-0.12	0.33	0.00
**5** (ME)	1.22	-0.64	-0.98	-0.28	0.68	0.00
**5** (EE)	0.40	-0.49	-0.10	-0.29	0.48	0.00

4. XYZ coordinates of QM atoms

ONIOM results

=== 1 (doublet) ===

h	39.045153	39.218807	22.616954
C	38.874503	39.129681	21.525052
H	37.837535	38.850650	21.334097
H	39.060852	40.116645	21.080422
S	40.034265	38.019763	20.693756
N	40.831128	35.832054	18.926637
C	41.182232	36.234591	17.663685
C	41.984839	35.349264	19.518782
C	42.592340	36.032689	17.456731
C	43.096637	35.497648	18.599200
C	41.998118	34.770999	20.780236
H	42.884630	34.230765	21.083538
C	40.963347	34.801036	21.695730
N	39.721172	35.381247	21.493494
C	39.042246	35.272857	22.696776
C	39.889962	34.628616	23.667894
C	41.060577	34.327650	23.050452
C	37.735507	35.675623	22.909741
H	37.332790	35.554408	23.909807
C	36.886590	36.215669	21.952038
C	35.527992	36.646859	22.194283
C	36.119191	37.042060	20.053152
N	37.229175	36.466022	20.639538
C	35.064225	37.164601	21.029687
C	36.017098	37.437192	18.729285
H	35.074123	37.885429	18.435842
C	37.013268	37.325678	17.766132
C	36.901696	37.762142	16.387094
N	38.280625	36.850117	18.013558
C	38.974640	36.992909	16.834097
C	40.328640	36.729373	16.693527
H	40.792297	36.914186	15.732876
Fe	38.957919	35.962635	19.718306
C	38.117926	37.554657	15.806921
h	38.457510	37.749773	14.867599
h	44.082922	35.294899	18.711774
h	35.972873	38.123501	15.936070
h	43.160131	36.462543	16.626358
h	41.926964	33.804022	23.458806
h	34.086904	37.609249	20.845651
h	34.971757	36.515534	23.126616
h	39.686694	34.603533	24.735722
O	38.490885	34.454950	19.340966
C	38.663752	26.475103	23.714926
C	37.540124	27.400755	23.329753
O	36.671440	27.791759	24.083762
O	37.637440	27.730504	22.030211
C	36.616527	28.574087	21.458695
C	35.399179	27.763611	21.067952
C	34.167592	28.041326	21.554395
C	33.862189	28.903696	22.748810
C	32.967757	27.450182	20.819946
O	31.766697	28.244845	21.068839
C	30.914193	27.854016	22.053994
O	31.081093	26.891204	22.768201
C	29.783702	28.889374	22.178336
O	30.274303	29.915445	23.053244
C	28.460329	28.343044	22.743766
N	28.552428	28.212664	24.195988
C	27.649772	27.414251	24.851309
O	26.961446	26.587490	24.257305
C	27.564068	27.585696	26.339820
C	28.496192	28.303239	27.100437
C	28.324030	28.433732	28.476900
C	27.226353	27.844475	29.103922
C	26.299449	27.119943	28.353756
C	26.471379	26.990509	26.979502
C	27.250680	29.205117	22.368182
C	26.116184	28.602542	21.812039
C	24.967852	29.351711	21.546965
C	24.934778	30.713718	21.847057
C	26.059643	31.324305	22.405909
C	27.208081	30.576845	22.666322
C	33.168788	27.430581	19.285676
C	34.632921	27.187241	18.796273
O	34.623595	26.122156	17.849183
C	35.310470	28.443339	18.106678
O	34.752856	28.587562	16.781280
C	35.500352	28.446617	15.640867
O	35.094717	28.979471	14.631729
C	36.803601	27.697809	15.628306
C	37.922338	28.416431	15.187133
C	39.150846	27.780143	15.051083
C	39.270871	26.414503	15.302519
C	38.150441	25.684328	15.697475
C	36.920663	26.322279	15.871390
C	35.223460	29.818338	18.881951
C	34.388411	30.937710	18.203407
O	32.973134	30.639958	18.400866
C	32.400370	30.982105	19.571701
C	30.906056	30.901725	19.475138
O	33.018585	31.359555	20.550388
C	34.570780	31.394819	16.738595
O	34.951102	32.724758	17.145873
C	34.697789	32.431595	18.539631
C	35.861137	32.791406	19.446733
C	36.399778	31.615665	20.256251
O	37.564820	32.002685	20.962608
C	36.626847	30.333049	19.359108
C	37.552837	30.668533	18.175501
C	37.355708	29.328046	20.291128
O	38.549653	29.169078	20.187331
C	35.532344	26.681551	19.976243
C	35.022706	25.305497	20.508953
C	36.978154	26.363842	19.515298
H	38.277940	25.752776	24.433401
H	39.083705	25.972265	22.842916
H	39.466879	27.045399	24.193922
H	36.343019	29.332987	22.196837
H	34.745149	29.037370	23.371202
H	33.446385	29.873943	22.455543
H	33.107612	28.409547	23.368178
H	32.737750	26.454749	21.193474
H	29.574723	29.291214	21.184145
H	30.682693	30.633087	22.532854
H	28.306614	27.335516	22.349125
H	28.909517	29.035985	24.660671
H	29.373882	28.740301	26.633750
H	29.043316	28.993556	29.064948
H	27.098296	27.939579	30.177602
H	25.449999	26.650268	28.843156
H	25.776420	26.424685	26.371495
H	26.125360	27.537586	21.603297
H	24.100992	28.869518	21.102028
H	24.043269	31.299413	21.640597
H	26.043500	32.386778	22.634309
H	28.079144	31.062199	23.095567
H	32.790681	28.364965	18.866926
H	32.552411	26.632438	18.857504
H	34.020444	26.372500	17.102677
H	36.359056	28.186536	17.972078
H	37.825292	29.472699	14.956150
H	40.010657	28.356123	14.736488
H	40.236007	25.933030	15.186207
H	38.230678	24.616849	15.877694
H	36.059909	25.759600	16.207161
H	34.664162	29.625726	19.791120
H	30.533291	31.672378	18.795867
H	30.587362	29.939967	19.072418
H	30.452918	31.062355	20.450717
H	33.634916	31.376150	16.175488
H	35.349172	30.924399	16.142285
H	33.801989	32.952710	18.880347
H	36.659428	33.203524	18.823329
H	35.575351	33.580202	20.148881
H	35.660090	31.343071	21.014480
H	38.058908	32.636256	20.405665
H	38.329351	31.373321	18.467706
H	38.054071	29.782967	17.778393
H	36.992725	31.147404	17.373897
H	33.947865	25.151425	20.415084

H	35.295281	25.197828	21.561602
H	35.503277	24.502571	19.952622
H	37.514513	25.849425	20.314363

=== 2a* (doublet) ===

h	38.858026	39.033595	22.337887
C	38.642822	38.945283	21.251126
H	37.588798	38.700916	21.109611
H	38.822598	39.923212	20.791448
S	39.732586	37.786847	20.356466
N	40.543788	35.554864	18.905551
C	40.869783	35.934665	17.628515
C	41.720431	35.128688	19.503263
C	42.280007	35.748176	17.405866
C	42.816818	35.283086	18.565307
C	41.761205	34.560402	20.765502
H	42.676282	34.072510	21.072222
C	40.717831	34.532957	21.675031
N	39.454322	35.048370	21.483335
C	38.826044	35.012074	22.706190
C	39.709575	34.424723	23.686191
C	40.865388	34.115647	23.047411
C	37.541250	35.467536	22.953600
H	37.171879	35.398655	23.971132
C	36.695018	36.036167	22.014788
C	35.376932	36.546972	22.286343
C	35.913485	36.904751	20.124204
N	37.003945	36.254572	20.679295
C	34.907023	37.087136	21.132402
C	35.788462	37.326299	18.809799
H	34.864761	37.834231	18.556916
C	36.752370	37.185798	17.824310
C	36.650911	37.684244	16.465756
N	37.986033	36.612301	18.026807
C	38.676171	36.763302	16.844948
C	40.011748	36.447205	16.672906
H	40.466448	36.638180	15.709621
Fe	38.690596	35.757255	19.755961
C	37.839377	37.420779	15.855652
h	38.175972	37.640565	14.920394
h	43.811854	35.127889	18.685594
h	35.752249	38.152252	16.055401
h	42.806952	36.157998	16.538190
h	41.746404	33.610685	23.448889
h	33.963298	37.610576	20.986333
h	34.840987	36.464364	23.233902
h	39.553970	34.482928	24.761467
O	38.214436	34.108902	19.270770
C	38.697079	26.401292	23.710749
C	37.595386	27.352918	23.330447
O	36.721657	27.746820	24.076576
O	37.723495	27.708982	22.039942
C	36.740788	28.596993	21.472942
C	35.500306	27.838971	21.056278
C	34.273074	28.140477	21.538849
C	33.969648	28.980520	22.747216
C	33.067531	27.591452	20.782284
O	31.876568	28.391917	21.049122
C	31.015069	27.979601	22.016467
O	31.172381	26.997202	22.705716
C	29.886036	29.013721	22.158791
O	30.388920	30.008866	23.064746
C	28.558434	28.448082	22.698479
N	28.638390	28.297570	24.149495
C	27.730315	27.490007	24.786630
O	27.051948	26.667208	24.176118
C	27.624419	27.648539	26.275328
C	28.546285	28.358468	27.055472
C	28.352721	28.480112	28.430000
C	27.243821	27.889541	29.035775
C	26.327475	27.172014	28.266212
C	26.520799	27.051102	26.893949
C	27.345332	29.304646	22.323706
C	26.221239	28.699816	21.749231
C	25.068573	29.441995	21.484229
C	25.020668	30.799668	21.801347
C	26.134682	31.412700	22.378917
C	27.286992	30.671477	22.640413
C	33.280764	27.631430	19.251767
H	36.921065	25.698020	18.652681
H	37.591427	27.214867	19.231956
C	34.738233	27.345561	18.768818
O	34.693257	26.300371	17.800469
C	35.465147	28.597650	18.109370
O	34.923804	28.787333	16.786189
C	35.658027	28.628832	15.637769
O	35.263241	29.198090	14.643388
C	36.924499	27.821113	15.584584
C	38.029337	28.467594	15.013720
C	39.216200	27.774195	14.809387
C	39.309275	26.420207	15.126969
C	38.201485	25.760670	15.656609
C	37.014076	26.456232	15.893369
C	35.419845	29.962349	18.917181
C	34.651396	31.145837	18.260300
O	33.227281	30.897153	18.404300
C	32.629402	31.205559	19.572625
C	31.138196	31.081511	19.452755
O	33.230044	31.580119	20.562781
C	34.910824	31.655849	16.824773
O	35.476220	32.889338	17.317169
C	35.044291	32.617029	18.664609
C	36.072704	32.867249	19.743336
C	36.671548	31.629294	20.385929
O	37.850460	31.938504	21.096835
C	36.841147	30.400114	19.408371
C	37.775299	30.726342	18.218275
C	37.521123	29.332121	20.321068
O	38.703587	29.110847	20.219914
C	35.606215	26.776095	19.943053
C	35.036139	25.407005	20.441036
C	37.043014	26.413528	19.487319
H	38.302040	25.696390	24.440092
H	39.092938	25.881553	22.837426
H	39.522596	26.954257	24.171507
H	36.488399	29.353858	22.220655
H	34.857384	29.135195	23.357429
H	33.513396	29.937808	22.472546
H	33.240951	28.455018	23.372515
H	32.825942	26.584998	21.118754
H	29.689964	29.445917	21.174475
H	30.638543	30.815074	22.573441
H	28.417482	27.445338	22.287097
H	28.989082	29.116588	24.626611
H	29.431350	28.798192	26.605398
H	29.063547	29.034471	29.033178
H	27.098618	27.978051	30.107899
H	25.469508	26.701311	28.739324
H	25.834113	26.491166	26.271206
H	26.241812	27.637889	21.526812
H	24.210048	28.957499	21.026001
H	24.125726	31.379876	21.594457
H	26.105659	32.471329	22.622545
H	28.147369	31.159299	23.089017
H	32.948403	28.599163	18.871895
H	32.632379	26.882601	18.782885
H	34.070136	26.574370	17.077504
H	36.507849	28.313315	17.978339
H	37.949363	29.511888	14.729142
H	40.063738	28.294989	14.385336
H	40.240849	25.892034	14.952580
H	38.257371	24.702057	15.889506
H	36.162422	25.947036	16.322760
H	34.838063	29.772826	19.813858
H	30.745407	31.854762	18.787677
H	30.857077	30.118828	19.023011
H	30.669357	31.196975	20.426985
H	33.975603	31.808157	16.275697
H	35.607374	31.123446	16.182319
H	34.152751	33.207267	18.894756
H	37.086478	33.594943	19.286854
H	35.719974	33.556624	20.512707
H	35.941202	31.309134	21.139863
H	38.362398	32.559844	20.536538
H	38.302526	31.671544	18.362438
H	38.521328	29.942836	18.075068
H	37.217682	30.830107	17.289889
H	33.978306	25.255032	20.225690

H	35.190626	25.311816	21.518132
H	35.571372	24.592445	19.955968
H	37.561039	25.886457	20.290164
H	36.966627	25.745127	18.628530
H	37.682974	27.242531	19.199038

=== 2b* (doublet) ===

h	38.860449	38.981324	22.517197
C	38.640960	38.839507	21.438718
H	37.595842	38.556241	21.310487
H	38.804775	39.803647	20.939713
S	39.764750	37.692908	20.596977
N	40.553320	35.526522	18.872392
C	40.883015	35.933274	17.608378
C	41.731539	35.108272	19.461612
C	42.298224	35.766458	17.380251
C	42.834983	35.280107	18.529235
C	41.776032	34.560922	20.737636
H	42.684179	34.058339	21.042988
C	40.750467	34.578660	21.665359
N	39.488917	35.122655	21.471682
C	38.846173	35.056937	22.697541
C	39.731593	34.471589	23.672679
C	40.889345	34.160627	23.034703
C	37.541049	35.458808	22.925498
H	37.170237	35.384835	23.942829
C	36.666309	35.977195	21.976386
C	35.342435	36.482982	22.262351
C	35.868125	36.821086	20.097292
N	36.963761	36.178807	20.642996
C	34.860934	37.011706	21.110660
C	35.753958	37.247650	18.784198
H	34.825762	37.744800	18.524532
C	36.730719	37.130096	17.801551
C	36.626428	37.631570	16.446818
N	37.978357	36.589522	18.015508
C	38.669016	36.755262	16.835161
C	40.015345	36.460378	16.667614
H	40.468941	36.672195	15.707409
Fe	38.674064	35.710618	19.705166
C	37.827017	37.397992	15.843350
h	38.164022	37.628337	14.910711
h	43.830077	35.123842	18.645928
h	35.721861	38.084542	16.032545
h	42.824884	36.197242	16.522946
h	41.768315	33.653402	23.437218
h	33.927635	37.556113	20.974978
h	34.821926	36.415126	23.219781
h	39.564765	34.499753	24.747380
O	38.221827	34.055417	19.313328
C	38.672993	26.382436	23.721418
C	37.566470	27.328131	23.339183
O	36.694977	27.723385	24.087563
O	37.687785	27.675817	22.046381
C	36.705301	28.565644	21.480104
C	35.463567	27.809219	21.064753
C	34.237466	28.113143	21.548423
C	33.937416	28.955799	22.755611
C	33.029612	27.567980	20.792608
O	31.841499	28.372240	21.061706
C	30.979787	27.964442	22.029854
O	31.129442	26.978647	22.716231
C	29.861852	29.009695	22.178662
O	30.378976	29.996162	23.085571
C	28.528965	28.458552	22.719436
N	28.608560	28.299450	24.169695
C	27.692153	27.497875	24.801986
O	27.005488	26.685857	24.186159
C	27.584712	27.647860	26.291565
C	28.506867	28.349940	27.078262
C	28.309875	28.464228	28.452911
C	27.197189	27.874413	29.052232
C	26.280517	27.164422	28.276215
C	26.477326	27.050674	26.903926
C	27.325683	29.331847	22.350831
C	26.196593	28.743927	21.768732
C	25.051961	29.499941	21.508416
C	25.017230	30.855296	21.836501
C	26.136414	31.451722	22.421501
C	27.280766	30.696614	22.679011
C	33.241324	27.609045	19.261934
C	34.697887	27.321141	18.777377
O	34.650165	26.276624	17.808085
C	35.425883	28.573120	18.119468
O	34.882490	28.764257	16.796583
C	35.614429	28.604035	15.648335
O	35.220985	29.171800	14.652458
C	36.878965	27.792699	15.593927
C	37.989649	28.440638	15.036700
C	39.173611	27.743565	14.827638
C	39.256954	26.384445	15.125526
C	38.143493	25.724196	15.642432
C	36.959447	26.423471	15.885082
C	35.382739	29.936902	18.928579
C	34.615930	31.121984	18.273077
O	33.190002	30.871489	18.412053
C	32.587068	31.176777	19.577823
C	31.095801	31.056042	19.449740
O	33.180836	31.544019	20.574709
C	34.880787	31.635249	16.840572
O	35.451000	32.863268	17.336767
C	35.011316	32.589597	18.687336
C	36.026279	32.833381	19.770260
C	36.633380	31.598599	20.406177
O	37.821057	31.905679	21.109005
C	36.804314	30.373229	19.421485
C	37.740028	30.705863	18.234232
C	37.484791	29.302101	20.327918
O	38.666944	29.077931	20.225016
C	35.565754	26.748030	19.949870
C	34.992822	25.379588	20.446122
C	37.001079	26.383246	19.491497
H	38.279991	25.674905	24.449372
H	39.073907	25.865311	22.848864
H	39.493615	26.940207	24.185401
H	36.454893	29.321829	22.228748
H	34.826210	29.109294	23.364630
H	33.484344	29.913865	22.478765
H	33.207303	28.434087	23.382388
H	32.784819	26.561999	21.128058
H	29.668177	29.446766	21.196208
H	30.629144	30.803464	22.595558
H	28.374217	27.459745	22.303630
H	28.972132	29.110122	24.651346
H	29.394253	28.790135	26.633313
H	29.020557	29.012629	29.061549
H	27.049184	27.957894	30.124317
H	25.419377	26.694317	28.744160
H	25.790236	26.496929	26.276153
H	26.207129	27.684113	21.535732
H	24.189416	29.027921	21.044971
H	24.128328	31.445841	21.632418
H	26.116728	32.508249	22.674540
H	28.145286	31.171675	23.133384
H	32.910978	28.577986	18.883552
H	32.591068	26.861950	18.792863
H	34.029351	26.553427	17.084330
H	36.468021	28.287596	17.986136
H	37.916191	29.489114	14.766099
H	40.026162	28.265686	14.415053
H	40.185331	25.852680	14.945281
H	38.191994	24.661923	15.859759
H	36.103697	25.913589	16.305513
H	34.800633	29.747797	19.825129
H	30.709170	31.819600	18.770354
H	30.814571	30.087453	19.033289
H	30.621527	31.187143	20.419496
H	33.946863	31.791155	16.289429
H	35.575594	31.100180	16.197938
H	34.117599	33.181345	18.906534
H	37.135717	33.635004	19.327251
H	35.675544	33.524553	20.537872
H	35.917020	31.265246	21.167952
H	38.328262	32.524988	20.541527

H	38.263497	31.652368	18.383653
H	38.488859	29.924915	18.090767
H	37.184519	30.811275	17.304820
H	33.934384	25.230648	20.231521
H	35.148106	25.282408	21.522912
H	35.525667	24.564511	19.959364
H	37.518150	25.851358	20.291856
H	36.921922	25.718857	18.629725
H	37.642805	27.212041	19.206681

=== 3a (doublet) ===

h	38.966030	39.157294	22.355213
C	38.787518	39.122075	21.258888
H	37.735651	38.906301	21.070808
H	39.015741	40.111778	20.849391
S	39.881730	37.968282	20.365077
N	40.735721	35.757286	18.939937
C	41.066949	36.117408	17.658161
C	41.891109	35.266361	19.529217
C	42.471281	35.895148	17.438527
C	42.989058	35.391029	18.589712
C	41.902357	34.659547	20.773679
H	42.798124	34.133271	21.074127
C	40.847136	34.637244	21.671567
N	39.603710	35.195130	21.478761
C	38.944005	35.120774	22.684018
C	39.790844	34.466513	23.653617
C	40.953900	34.157807	23.027452
C	37.663076	35.590234	22.928824
H	37.269009	35.471603	23.932301
C	36.837741	36.202249	21.998525
C	35.490564	36.653942	22.245639
C	36.082965	37.090625	20.111397
N	37.182621	36.485244	20.688677
C	35.036132	37.208145	21.092313
C	35.970117	37.494747	18.788944
H	35.044319	37.988178	18.515429
C	36.935231	37.324511	17.808325
C	36.828824	37.766318	16.431646
N	38.172800	36.751402	18.023946
C	38.862739	36.871677	16.834655
C	40.206975	36.580976	16.678993
H	40.663888	36.740322	15.710636
Fe	38.912079	36.011152	19.789126
C	38.016499	37.479957	15.826326
h	38.347569	37.654217	14.879826
h	43.975681	35.188339	18.700321
h	35.920414	38.198759	16.002196
h	43.020113	36.307830	16.586570
h	41.815554	33.619339	23.427054
h	34.053166	37.639831	20.908592
h	34.925143	36.504402	23.168986
h	39.605201	34.471496	24.725661
O	38.301118	34.342044	19.346996
C	38.673736	26.362468	23.703635
C	37.553845	27.298419	23.336851
O	36.689196	27.684953	24.097620
O	37.649791	27.647383	22.041613
C	36.640441	28.518108	21.493821
C	35.414419	27.736303	21.079745
C	34.184468	28.018236	21.567215
C	33.879569	28.856337	22.778326
C	32.981827	27.457747	20.813969
O	31.787925	28.257894	21.070938
C	30.924618	27.856790	22.042377
O	31.083102	26.885247	22.746466
C	29.796539	28.894579	22.161625
O	30.288242	29.923298	23.031958
C	28.470733	28.353418	22.724376
N	28.557809	28.222245	24.176820
C	27.653984	27.419077	24.825775
O	26.971728	26.591780	24.225734
C	27.559759	27.586589	26.313880
C	28.492092	28.295280	27.082503
C	28.311502	28.423100	28.458139
C	27.205725	27.839604	29.076205
C	26.278875	27.123452	28.318021
C	26.458839	26.996928	26.944570
C	27.265003	29.218153	22.342692
C	26.133612	28.618431	21.777124
C	24.988916	29.370246	21.503889
C	24.956293	30.731958	21.805376
C	26.078059	31.339669	22.373566
C	27.223194	30.589756	22.641636
C	33.190425	27.472968	19.281692
C	34.653780	27.230802	18.793794
O	34.633095	26.194425	17.814913
C	35.348464	28.502924	18.144188
O	34.809916	28.685882	16.817208
C	35.560518	28.562091	15.676250
O	35.163914	29.131741	14.682833
C	36.847418	27.786880	15.631233
C	37.952775	28.471132	15.107286
C	39.158851	27.807355	14.917031
C	39.270944	26.447178	15.200892
C	38.163449	25.750908	15.682275
C	36.956415	26.416306	15.905931
C	35.260274	29.861127	18.954955
C	34.433421	31.003526	18.294603
O	33.017962	30.693468	18.426843
C	32.406417	30.994853	19.591632
C	30.915997	30.927419	19.446400
O	32.996577	31.330352	20.601682
C	34.698411	31.545913	16.871970
O	35.208702	32.795346	17.391088
C	34.745223	32.462062	18.742223
C	35.718307	32.689557	19.814140
C	36.491365	31.581086	20.424510
O	37.707986	32.039314	20.969873
C	36.673479	30.340977	19.442810
C	37.602147	30.708052	18.263045
C	37.392514	29.298768	20.350037
O	38.587905	29.146504	20.265711
C	35.539404	26.680237	19.963103
C	35.007451	25.298607	20.459753
C	36.984237	26.356664	19.503379
H	38.290880	25.642316	24.424793
H	39.077818	25.859326	22.824397
H	39.488580	26.923755	24.173315
H	36.374193	29.254179	22.256998
H	34.759765	28.979445	23.407158
H	33.461292	29.830550	22.502687
H	33.123245	28.349328	23.384812
H	32.741035	26.455779	21.163335
H	29.591235	29.291756	21.164472
H	30.706112	30.633090	22.508472
H	28.315600	27.345950	22.330230
H	28.905780	29.047602	24.644961
H	29.375410	28.728145	26.622482
H	29.030205	28.976591	29.052620
H	27.071111	27.932605	30.149221
H	25.423290	26.658089	28.800697
H	25.763948	26.437592	26.330538
H	26.142340	27.553746	21.566735
H	24.124554	28.890468	21.051536
H	24.067608	31.319690	21.592562
H	26.062149	32.401957	22.602817
H	28.092263	31.073172	23.077146
H	32.820696	28.418502	18.881469
H	32.569571	26.689922	18.832104
H	34.005887	26.464889	17.094465
H	36.398647	28.246610	18.016548
H	37.859738	29.521330	14.849573
H	40.007371	28.356086	14.531667
H	40.218308	25.943175	15.040396
H	38.235164	24.687664	15.889276
H	36.104999	25.879654	16.301448
H	34.690543	29.653710	19.854980
H	30.567950	31.721824	18.781454
H	30.604385	29.981407	19.002810
H	30.433843	31.058868	20.412472
H	33.769220	31.673233	16.306980
H	35.424196	31.046401	16.235502
H	33.834234	33.029105	18.954129

```
H    37.708812   34.382916   18.576349
H    35.785313   33.664985   20.283825
H    35.906733   31.196121   21.272639
H    38.047128   32.756379   20.386374
H    38.148828   31.630088   18.465879
H    38.340155   29.925516   18.076889
H    37.041112   30.875009   17.346434
H    33.935986   25.150550   20.324892
H    35.242458   25.174710   21.519599
H    35.504982   24.500989   19.911057
H    37.510042   25.819388   20.294267
H    36.925892   25.709395   18.627021
H    37.608579   27.206808   19.240885

=== 3b (doublet) ===
h    38.980192   39.144160   22.521159
C    38.797149   39.039500   21.431459
H    37.751417   38.786810   21.255050
H    39.012553   40.009062   20.963809
S    39.923758   37.862614   20.638041
N    40.737767   35.730039   18.869658
C    41.077179   36.125791   17.602908
C    41.898783   35.258037   19.456450
C    42.488853   35.934872   17.386176
C    43.004766   35.411538   18.529440
C    41.918348   34.673753   20.716791
H    42.811900   34.143993   21.018717
C    40.879660   34.684566   21.630966
N    39.635641   35.249952   21.423392
C    38.962496   35.161156   22.629375
C    39.813104   34.525781   23.608277
C    40.982143   34.221005   22.991077
C    37.661688   35.585480   22.844143
H    37.265367   35.474750   23.848510
C    36.811895   36.154643   21.899669
C    35.467787   36.617162   22.165621
C    36.040957   37.037958   20.025383
N    37.140863   36.415602   20.585051
C    35.002491   37.169497   21.016643
C    35.942416   37.459066   18.707335
H    35.015339   37.949122   18.429445
C    36.924137   37.315158   17.731636
C    36.821158   37.763124   16.355670
N    38.167988   36.769200   17.964550
C    38.858677   36.889172   16.779538
C    40.210329   36.607074   16.634921
H    40.668301   36.781447   15.669336
Fe   38.883467   35.954249   19.679491
C    38.017930   37.491516   15.761049
h    38.356604   37.670474   14.818231
h    43.994035   35.220215   18.636950
h    35.912727   38.190449   15.921496
h    43.052244   36.375358   16.557722
h    41.849226   33.699328   23.400006
h    34.031426   37.634327   20.853569
h    34.920164   36.491855   23.103570
h    39.616695   34.519113   24.678105
O    38.302025   34.279564   19.259062
C    38.684140   26.385795   23.732309
C    37.560413   27.314539   23.358802
O    36.695935   27.705080   24.117860
O    37.652872   27.651845   22.060477
C    36.644812   28.521836   21.508534
C    35.419302   27.738907   21.095785
C    34.189034   28.020519   21.582614
C    33.882118   28.860293   22.791639
C    32.986669   27.458486   20.830069
O    31.792071   28.258514   21.084985
C    30.927778   27.860592   22.056377
O    31.085432   26.892025   22.764813
C    29.798470   28.897974   22.168414
O    30.287288   29.932883   23.032887
C    28.472346   28.358884   22.731982
N    28.557000   28.233963   24.185146
C    27.652375   27.433065   24.835573
O    26.971281   26.603367   24.237453
C    27.555347   27.605522   26.322923
C    28.484992   28.318416   27.090884
C    28.302274   28.449262   28.465947
C    27.196918   27.865003   29.084005
C    26.272451   27.145161   28.326428
C    26.454637   27.015409   26.953597
C    27.266496   29.221133   22.344967
C    26.136374   28.618147   21.780301
C    24.991355   29.367820   21.502549
C    24.957083   30.730670   21.798654
C    26.077586   31.341647   22.365833
C    27.223100   30.593895   22.638326
C    33.195638   27.472438   19.298105
C    34.659288   27.231946   18.811105
O    34.639280   26.195762   17.831893
C    35.353809   28.504620   18.162558
O    34.815330   28.688932   16.835491
C    35.564815   28.559789   15.694331
O    35.169110   29.127449   14.699490
C    36.849479   27.780105   15.650147
C    37.959476   28.462707   15.134120
C    39.163821   27.795283   14.944931
C    39.269339   26.433013   15.220965
C    38.157192   25.738470   15.694070
C    36.952064   26.407487   15.917168
C    35.268522   29.863677   18.973279
C    34.438875   31.003331   18.313590
O    33.023402   30.688044   18.437830
C    32.403345   30.986635   19.598699
C    30.913853   30.917604   19.443164
O    32.985231   31.320483   20.614157
C    34.710315   31.553235   16.895297
O    35.214034   32.801097   17.424682
C    34.742040   32.460225   18.770839
C    35.707557   32.683752   19.850162
C    36.520212   31.586981   20.431839
O    37.744791   32.064727   20.937243
C    36.686189   30.342263   19.451645
C    37.611743   30.694118   18.263606
C    37.401339   29.300903   20.364615
O    38.597307   29.149727   20.289326
C    35.544864   26.681898   19.980450
C    35.013160   25.300617   20.478331
C    36.989892   26.359002   19.520968
H    38.302661   25.665681   24.454224
H    39.094408   25.882373   22.856141
H    39.493584   26.953323   24.203940
H    36.377549   29.258819   22.270304
H    34.762959   28.993500   23.417462
H    33.454573   29.829543   22.513311
H    33.131553   28.349086   23.401756
H    32.746045   26.456797   21.180357
H    29.594011   29.288683   21.168520
H    30.710362   30.636527   22.505241
H    28.318318   27.349621   22.342030
H    28.904684   29.060984   24.650547
H    29.367949   28.752177   26.631021
H    29.019077   29.005588   29.060073
H    27.060877   27.960260   30.156623
H    25.417114   26.679304   28.809094
H    25.761774   26.453020   26.340041
H    26.146395   27.552653   21.574118
H    24.128060   28.885530   21.050811
H    24.068162   31.316744   21.582254
H    26.060546   32.404897   22.590506
H    28.091425   31.079810   23.072503
H    32.825239   28.417106   18.896692
H    32.575835   26.688230   18.849020
H    34.009210   26.465082   17.113521
H    36.403686   28.247599   18.034153
H    37.871693   29.514746   14.882197
H    40.016306   28.342953   14.566747
H    40.215136   25.925981   15.060655
H    38.223607   24.673640   15.894661
H    36.097492   25.871657   16.306671
H    34.702286   29.658389   19.876082
H    30.569576   31.709283   18.773193
H    30.606158   29.969780   19.000712
```

H	30.424763	31.051982	20.405401
H	33.783952	31.680610	16.325371
H	35.441393	31.059433	16.260439
H	33.827931	33.023230	18.979456
H	37.484159	34.325866	18.734587
H	35.737572	33.643468	20.355996
H	35.968691	31.198485	21.300313
H	38.069419	32.776410	20.331491
H	38.120418	31.646249	18.423851
H	38.380435	29.932311	18.121645
H	37.054189	30.788553	17.334156
H	33.942371	25.150809	20.339708
H	35.244354	25.179281	21.539326
H	35.513953	24.502583	19.933264
H	37.516064	25.822575	20.312215
H	36.932200	25.711576	18.644616
H	37.613522	27.209642	19.258233

=== 4a* (doublet) ===

h	38.801274	38.982958	22.267141
C	38.575520	38.893963	21.181859
H	37.515059	38.676874	21.052229
H	38.782298	39.863600	20.717217
S	39.630267	37.696693	20.285833
N	40.413699	35.469326	18.888931
C	40.731657	35.831609	17.607280
C	41.594405	35.062503	19.484411
C	42.144774	35.653378	17.376515
C	42.690616	35.213266	18.540249
C	41.635147	34.498778	20.749728
H	42.554339	34.019816	21.058809
C	40.590047	34.459350	21.659078
N	39.318281	34.966343	21.473732
C	38.709464	34.950130	22.708466
C	39.601434	34.366736	23.681688
C	40.750430	34.050734	23.031729
C	37.437304	35.434936	22.974237
H	37.081331	35.374080	23.997211
C	36.593183	36.027281	22.048008
C	35.281284	36.554961	22.330403
C	35.812656	36.903175	20.165577
N	36.899155	36.246765	20.715225
C	34.811651	37.100254	21.180697
C	35.686002	37.318309	18.848933
H	34.775948	37.854271	18.602882
C	36.630939	37.140331	17.848001
C	36.532111	37.647657	16.494849
N	37.843523	36.510072	18.029479
C	38.526734	36.649144	16.835846
C	39.858434	36.318943	16.651089
H	40.302298	36.500809	15.680894
Fe	38.580804	35.760948	19.792226
C	37.699846	37.337451	15.863276
h	38.030057	37.550619	14.924242
h	43.687613	35.070697	18.660484
h	35.648037	38.152746	16.098014
h	42.662212	36.056475	16.500101
h	41.633588	33.545001	23.428529
h	33.869948	37.626798	21.037557
h	34.750975	36.473496	23.280286
h	39.464213	34.443601	24.758612
O	37.957924	34.016417	19.303821
C	38.685095	26.324991	23.754650
C	37.581557	27.271359	23.371075
O	36.719981	27.688029	24.118628
O	37.693684	27.598231	22.070733
C	36.734168	28.513318	21.511018
C	35.478958	27.792350	21.078578
C	34.256594	28.113261	21.560265
C	33.960463	28.943554	22.776404
C	33.045052	27.593660	20.792488
O	31.863665	28.403034	21.066909
C	30.997783	27.990055	22.029759
O	31.149334	27.003331	22.714002
C	29.872489	29.028058	22.171285
O	30.382850	30.019983	23.077108
C	28.543110	28.463237	22.707715
N	28.620905	28.311784	24.158597
C	27.714067	27.500209	24.792896
O	27.039445	26.676643	24.179355
C	27.604712	27.655858	26.281477
C	28.525218	28.363506	27.065329
C	28.327573	28.483287	28.439438
C	27.216365	27.892908	29.041106
C	26.301700	27.177223	28.267895
C	26.498863	27.058255	26.896025
C	27.330217	29.318233	22.329754
C	26.209577	28.711709	21.750259
C	25.056320	29.451601	21.481810
C	25.004392	30.808817	21.800177
C	26.114903	31.423645	22.382511
C	27.267833	30.684574	22.647518
C	33.262949	27.659731	19.264089
C	34.715087	27.354068	18.781543
O	34.653889	26.326267	17.795503
C	35.468001	28.605094	18.145628
O	34.940144	28.828154	16.822562
C	35.671786	28.673018	15.670431
O	35.284548	29.266964	14.687113
C	36.921553	27.841550	15.595053
C	38.014244	28.456434	14.966971
C	39.184388	27.742265	14.740539
C	39.272850	26.396760	15.094202
C	38.175974	25.767109	15.679342
C	37.005663	26.484139	15.936567
C	35.447764	29.959405	18.976824
C	34.719529	31.178113	18.331519
O	33.290409	30.955434	18.424246
C	32.668213	31.235163	19.589370
C	31.179347	31.109062	19.443087
O	33.252274	31.586210	20.597439
C	35.049783	31.728483	16.927288
O	35.690864	32.900494	17.491295
C	35.143262	32.618905	18.808876
C	36.099889	32.772095	19.935229
C	36.771513	31.558108	20.482891
O	37.991192	31.879438	21.105496
C	36.882472	30.341528	19.473680
C	37.818697	30.642380	18.277630
C	37.534546	29.243710	20.372183
O	38.713671	29.004880	20.277024
C	35.565760	26.745708	19.949410
C	34.960667	25.381924	20.424651
C	36.996342	26.359796	19.494162
H	38.295725	25.628220	24.494016
H	39.076249	25.796853	22.884497
H	39.512177	26.884383	24.204642
H	36.494658	29.263309	22.269671
H	34.850869	29.106444	23.380710
H	33.489447	29.896017	22.510765
H	33.242497	28.406877	23.404767
H	32.792768	26.583340	21.110113
H	29.680764	29.461317	21.186260
H	30.587168	30.843342	22.592925
H	28.403455	27.460394	22.296072
H	28.968485	29.131100	24.637500
H	29.411865	28.803458	26.618526
H	29.036731	29.036404	29.045532
H	27.067949	27.980202	30.112869
H	25.442045	26.706578	28.737920
H	25.813378	26.499916	26.270543
H	26.233359	27.650096	21.526626
H	24.200505	28.965644	21.020133
H	24.108939	31.387308	21.590843
H	26.082029	32.481694	22.627718
H	28.124823	31.174242	23.100927
H	32.951524	28.641205	18.902212
H	32.599793	26.934466	18.778863
H	34.023145	26.617001	17.085275
H	36.505996	28.303666	18.016623
H	37.936010	29.492470	14.654114
H	40.022878	28.239214	14.271911
H	40.191264	25.851602	14.903087
H	38.226802	24.714773	15.939873

```
H    36.160429   25.997320   16.402816
H    34.852797   29.774315   19.866279
H    30.788716   31.907115   18.806481
H    30.908600   30.163436   18.970261
H    30.699267   31.180046   20.416064
H    34.140199   31.979673   16.371220
H    35.717486   31.177336   16.270957
H    34.269689   33.253907   18.981593
H    37.620141   34.012695   18.390277
H    35.932412   33.573016   20.643237
H    36.104669   31.207560   21.285792
H    38.402926   32.551748   20.523475
H    38.304456   31.613490   18.379938
H    38.594182   29.880853   18.187315
H    37.274039   30.663033   17.336074
H    33.918598   25.232680   20.140233
H    35.045991   25.294501   21.509507
H    35.523319   24.560563   19.984211
H    37.506537   25.828127   20.298730
H    36.909146   25.689749   18.638049
H    37.648891   27.177545   19.201339
```

=== 4b* (doublet) ===

```
h    38.873080   39.029660   22.384558
C    38.659429   38.948436   21.297898
H    37.602841   38.719033   21.155398
H    38.854548   39.933572   20.856829
S    39.726984   37.818459   20.348433
N    40.490040   35.524522   18.855097
C    40.820900   35.915101   17.581805
C    41.671408   35.113726   19.450003
C    42.236611   35.757928   17.359402
C    42.771103   35.280431   18.513970
C    41.713693   34.572803   20.727146
H    42.623749   34.073138   21.031243
C    40.688922   34.587923   21.660786
N    39.430670   35.130441   21.483397
C    38.809166   35.075256   22.714301
C    39.695414   34.470607   23.679944
C    40.844351   34.159172   23.028966
C    37.517725   35.512059   22.966485
H    37.152770   35.431150   23.985372
C    36.655983   36.072787   22.031006
C    35.329270   36.575118   22.308527
C    35.879767   36.928412   20.150175
N    36.968494   36.288365   20.705675
C    34.860940   37.112421   21.155543
C    35.766284   37.339711   18.830119
H    34.850057   37.861816   18.575295
C    36.714626   37.169742   17.826305
C    36.589556   37.649963   16.463199
N    37.942174   36.575405   18.010562
C    38.600789   36.692968   16.805828
C    39.940966   36.385194   16.622455
H    40.381581   36.556806   15.647984
Fe   38.673195   35.829008   19.763764
C    37.754446   37.343016   15.824587
h    38.075006   37.538418   14.878894
h    43.767282   35.126022   18.621884
h    35.693701   38.135591   16.067407
h    42.781929   36.223408   16.530899
h    41.718760   33.632445   23.417332
h    33.909920   37.618772   21.000768
h    34.796970   36.484355   23.256843
h    39.542473   34.505644   24.756317
O    37.995306   34.057212   19.393206
C    38.683408   26.371209   23.758129
C    37.570509   27.306485   23.372655
O    36.708318   27.719710   24.121403
O    37.672787   27.625408   22.069552
C    36.697717   28.521955   21.506241
C    35.452584   27.777958   21.080961
C    34.227037   28.090137   21.560595
C    33.928141   28.930135   22.770537
C    33.019116   27.554560   20.798134
O    31.834825   28.364464   21.062598
C    30.971668   27.960466   22.032704
O    31.124940   26.979051   22.724050
C    29.849428   29.001882   22.173332
O    30.360249   29.995645   23.076545
C    28.518776   28.444721   22.713818
N    28.598537   28.294449   24.164512
C    27.691420   27.485488   24.801574
O    27.012112   26.664131   24.190246
C    27.588819   27.640846   26.290654
C    28.513284   28.348094   27.070240
C    28.323076   28.466566   28.445478
C    27.215237   27.875289   29.052454
C    26.296402   27.160403   28.283442
C    26.486202   27.042788   26.910429
C    27.310673   29.307144   22.336766
C    26.185922   28.707680   21.757968
C    25.037437   29.455083   21.489678
C    24.994531   30.812769   21.807409
C    26.109346   31.420551   22.388969
C    27.257565   30.674119   22.653789
C    33.236970   27.595123   19.268027
C    34.693783   27.302643   18.787788
O    34.645454   26.264787   17.811658
C    35.431400   28.553615   18.138131
O    34.901024   28.753859   16.812201
C    35.642431   28.609552   15.665279
O    35.252292   29.193301   14.677111
C    36.907791   27.802407   15.605244
C    37.999036   28.438972   14.996900
C    39.185608   27.746702   14.789769
C    39.291430   26.401992   15.141745
C    38.196331   25.750669   15.706578
C    37.009497   26.446246   15.946441
C    35.389048   29.914370   18.953622
C    34.626710   31.104669   18.296654
O    33.202924   30.862259   18.423347
C    32.598604   31.171398   19.590003
C    31.107745   31.060525   19.462709
O    33.198193   31.538521   20.583388
C    34.920858   31.629733   16.875672
O    35.542661   32.827267   17.401222
C    35.029582   32.560107   18.732111
C    35.989630   32.745692   19.845268
C    36.669630   31.567136   20.437671
O    37.866997   31.925956   21.073195
C    36.814858   30.332872   19.450800
C    37.756990   30.648337   18.265614
C    37.484708   29.258324   20.361795
O    38.667387   29.037430   20.266107
C    35.553570   26.720835   19.962220
C    34.971726   25.353888   20.451470
C    36.989851   26.350654   19.511669
H    38.297771   25.665874   24.491812
H    39.085853   25.851268   22.888122
H    39.500502   26.938231   24.216878
H    36.447815   29.273841   22.259832
H    34.815302   29.080473   23.382995
H    33.476885   29.890136   22.497095
H    33.195649   28.408160   23.394122
H    32.768466   26.549221   21.131447
H    29.655824   29.432567   21.187919
H    30.602293   30.804247   22.585455
H    28.372198   27.442288   22.303595
H    28.949666   29.112794   24.642399
H    29.397911   28.787873   26.619315
H    29.035673   29.018969   29.048293
H    27.072776   27.961177   30.125123
H    25.439368   26.689067   28.757573
H    25.797576   26.484812   26.288108
H    26.202679   27.645897   21.534292
H    24.178282   28.974549   21.028542
H    24.102761   31.396956   21.598108
H    26.083797   32.478986   22.633566
H    28.118347   31.158136   23.105859
H    32.909735   28.564481   18.887766
H    32.586135   26.849926   18.796842
H    34.022936   26.547887   17.091414
H    36.473274   28.264134   18.013049
```

H	37.907560	29.474185	14.685091
H	40.023507	28.260362	14.338496
H	40.223289	25.874726	14.966701
H	38.262281	24.698871	15.966098
H	36.165699	25.944602	16.400129
H	34.801926	29.724923	19.847007
H	30.722395	31.851055	18.813659
H	30.820291	30.109385	19.012029
H	30.637651	31.160119	20.437892
H	33.997640	31.843981	16.326480
H	35.592878	31.079474	16.223316
H	34.150346	33.184088	18.917471
H	38.221716	33.826100	18.478203
H	35.895063	33.618615	20.476243
H	35.978789	31.222513	21.226597
H	38.224743	32.674951	20.533128
H	38.288524	31.586434	18.425060
H	38.503732	29.863833	18.133563
H	37.209821	30.754167	17.331674
H	33.914456	25.209572	20.228397
H	35.119102	25.253965	21.528916
H	35.505743	24.537964	19.967835
H	37.502624	25.823157	20.317320
H	36.913400	25.680898	18.654274
H	37.634908	27.175889	19.223376

=== 5 (doublet) ===

h	38.991506	39.336747	22.382385
C	38.845875	39.319522	21.280841
H	37.799580	39.111693	21.054775
H	39.089102	40.313933	20.890928
S	39.967024	38.155631	20.427384
N	40.717219	35.898402	18.902010
C	41.063955	36.283678	17.630056
C	41.880990	35.407962	19.477318
C	42.469137	36.059943	17.400865
C	42.985791	35.540388	18.542923
C	41.887166	34.790988	20.715129
H	42.784111	34.270403	21.022796
C	40.826822	34.756313	21.605109
N	39.569967	35.303698	21.396290
C	38.921602	35.216492	22.617564
C	39.767062	34.557444	23.579639
C	40.935335	34.264956	22.953113
C	37.644301	35.684827	22.879367
H	37.258357	35.555563	23.885267
C	36.817862	36.312012	21.964415
C	35.465095	36.748430	22.226402
C	36.059070	37.210148	20.101940
N	37.170341	36.606646	20.659858
C	35.005932	37.311783	21.083417
C	35.946502	37.612435	18.782088
H	35.016900	38.096360	18.501404
C	36.918942	37.450932	17.806328
C	36.793776	37.868326	16.427193
N	38.177708	36.918475	18.038346
C	38.855492	37.049400	16.836059
C	40.201096	36.770000	16.667544
H	40.651637	36.950901	15.699449
Fe	38.959041	36.337545	19.811982
C	37.991000	37.616165	15.823054
h	38.313462	37.796206	14.874553
h	43.971184	35.331603	18.652882
h	35.862589	38.236179	15.986554
h	43.015123	36.465787	16.544223
h	41.808203	33.748670	23.358854
h	34.017346	37.731446	20.906310
h	34.906298	36.582873	23.151077
h	39.579004	34.544974	24.651862
O	37.101306	33.436158	19.249880
C	38.715229	26.366460	23.803062
C	37.569412	27.254482	23.404220
O	36.734006	27.708211	24.159634
O	37.604263	27.479235	22.077304
C	36.633983	28.380214	21.513434
C	35.389201	27.646454	21.075173
C	34.164356	27.959670	21.554880
C	33.873465	28.796739	22.770470
C	32.953516	27.434553	20.788998
O	31.777613	28.259544	21.046955
C	30.913485	27.874415	22.025169
O	31.061048	26.898249	22.724934
C	29.803255	28.929778	22.160015
O	30.321270	29.935332	23.044355
C	28.473287	28.393270	22.719754
N	28.565606	28.246833	24.170341
C	27.665139	27.435169	24.814304
O	26.986585	26.609114	24.208511
C	27.570178	27.593142	26.303273
C	28.494063	28.309193	27.075442
C	28.311572	28.428774	28.451497
C	27.212770	27.829444	29.066789
C	26.294423	27.106121	28.305124
C	26.476038	26.987936	26.931105
C	27.269892	29.266878	22.353739
C	26.129149	28.674173	21.799460
C	24.983333	29.431030	21.546638
C	24.959550	30.791378	21.855036
C	26.090978	31.392504	22.411027
C	27.236464	30.636759	22.661064
C	33.169531	27.457866	19.257160
C	34.627618	27.177931	18.774109
O	34.593258	26.143860	17.793883
C	35.349298	28.434373	18.128292
O	34.799692	28.655712	16.811876
C	35.538825	28.543576	15.659247
O	35.132667	29.127351	14.678341
C	36.830304	27.780028	15.602936
C	37.919987	28.475503	15.061056
C	39.140288	27.834933	14.886383
C	39.281138	26.484476	15.202547
C	38.188301	25.774797	15.698829
C	36.967241	26.418838	15.909059
C	35.314539	29.775569	18.961829
C	34.562770	30.977788	18.322756
O	33.133423	30.740607	18.423496
C	32.520288	31.038829	19.589666
C	31.030329	30.969442	19.443093
O	33.114777	31.371364	20.598597
C	34.865989	31.517948	16.906598
O	35.342502	32.781027	17.441558
C	34.923012	32.430652	18.779053
C	36.029217	32.691851	19.821465
C	36.550927	31.396155	20.457023
O	37.704546	31.681066	21.215991
C	36.738798	30.185738	19.461649
C	37.690849	30.488572	18.273546
C	37.421437	29.130082	20.385854
O	38.617342	28.969810	20.329154
C	35.495960	26.602728	19.946298
C	34.933964	25.228998	20.432640
C	36.935841	26.256306	19.489908
H	38.356531	25.656756	24.546406
H	39.138385	25.850195	22.941201
H	39.506286	26.970973	24.259351
H	36.385354	29.128268	22.270843
H	34.758715	28.925771	23.391106
H	33.447724	29.769416	22.500768
H	33.124474	28.288292	23.384762
H	32.690621	26.434419	21.128492
H	29.602935	29.346501	21.169608
H	30.656433	30.693582	22.529904
H	28.310348	27.390474	22.316900
H	28.909951	29.070053	24.644856
H	29.372813	28.754517	26.618500
H	29.023170	28.988709	29.048334
H	27.077064	27.916156	30.140205
H	25.444054	26.628984	28.785471
H	25.787354	26.423952	26.314304
H	26.131600	27.610748	21.582834
H	24.111352	28.955913	21.104356
H	24.069843	31.382710	21.657226
H	26.081564	32.453434	22.646306
H	28.111519	31.115209	23.090628

H	32.829718	28.418533	18.865131
H	32.527176	26.698896	18.796907
H	33.981944	26.426884	17.064099
H	36.389149	28.148599	17.984104
H	37.804778	29.517696	14.780144
H	39.976157	28.395641	14.490703
H	40.240229	25.998791	15.056802
H	38.283444	24.718608	15.931053
H	36.126373	25.874633	16.318125
H	34.727279	29.577635	19.852179
H	30.680499	31.755314	18.769238
H	30.721156	30.016777	19.011402
H	30.548940	31.107638	20.408465
H	33.962165	31.642651	16.305108
H	35.628385	31.032432	16.304134
H	34.034854	33.000662	19.051806
H	36.931672	33.455917	18.289405
H	35.621041	33.296498	20.636456
H	35.786191	31.079603	21.169685
H	38.283533	32.215061	20.641617
H	38.076201	31.507343	18.306979
H	38.542722	29.807858	18.276684
H	37.187483	30.370825	17.314573
H	33.866236	25.092296	20.260622
H	35.132549	25.108931	21.499903
H	35.443296	24.423430	19.908018
H	37.459226	25.730811	20.289656
H	36.868541	25.595041	18.625158
H	37.566878	27.095247	19.207255

=== 1 (quartet) ===

h	39.048639	39.226779	22.627556
C	38.879279	39.134869	21.535770
H	37.843769	38.850262	21.344274
H	39.060710	40.121965	21.089238
S	40.045155	38.028321	20.709438
N	40.835029	35.829759	18.929839
C	41.184689	36.232856	17.666191
C	41.990232	35.351334	19.520949
C	42.595223	36.033665	17.458484
C	43.101467	35.501064	18.601131
C	42.004640	34.777414	20.784337
H	42.891774	34.239891	21.090441
C	40.969363	34.810243	21.698264
N	39.722689	35.384230	21.492802
C	39.045326	35.278619	22.699328
C	39.895844	34.640580	23.670603
C	41.067361	34.341456	23.053531
C	37.737324	35.674970	22.913314
H	37.336115	35.555120	23.914107
C	36.886724	36.209655	21.954409
C	35.528230	36.641190	22.195787
C	36.121248	37.035200	20.054843
N	37.230631	36.456911	20.642098
C	35.065570	37.158478	21.030688
C	36.020125	37.434230	18.732641
H	35.077038	37.882271	18.439314
C	37.017301	37.325428	17.770004
C	36.903997	37.758957	16.390163
N	38.285450	36.854308	18.018324
C	38.977876	36.993221	16.837995
C	40.331533	36.728312	16.696535
H	40.794776	36.912337	15.735534
Fe	38.959386	35.948889	19.722765
C	38.120187	37.551541	15.809729
h	38.459007	37.745006	14.869797
h	44.088078	35.300104	18.713623
h	35.974157	38.117161	15.938695
h	43.161305	36.464271	16.627301
h	41.934828	33.820286	23.462857
h	34.089463	37.605956	20.846601
h	34.972239	36.512243	23.128545
h	39.692216	34.614352	24.738337
O	38.484659	34.456185	19.302165
C	38.666371	26.482328	23.715843
C	37.541944	27.406527	23.329519
O	36.674495	27.799889	24.083739
O	37.636641	27.731593	22.028571
C	36.615435	28.574409	21.456479
C	35.398129	27.763399	21.066681
C	34.166688	28.041246	21.553425
C	33.861727	28.904434	22.747392
C	32.966639	27.449399	20.819837
O	31.765554	28.244056	21.068634
C	30.913433	27.853766	22.054371
O	31.080768	26.891452	22.769145
C	29.782726	28.888939	22.178301
O	30.272946	29.915593	23.052696
C	28.459486	28.342644	22.744062
N	28.551691	28.212830	24.196316
C	27.649397	27.414293	24.851985
O	26.961177	26.587216	24.258305
C	27.563855	27.586196	26.340445
C	28.495936	28.304203	27.100676
C	28.324025	28.435011	28.477138
C	27.226617	27.845640	29.104525
C	26.299743	27.120676	28.354740
C	26.471440	26.990906	26.980491
C	27.249680	29.204368	22.368171
C	26.115310	28.601370	21.812228
C	24.966836	29.350220	21.546869
C	24.933491	30.712327	21.846481
C	26.058226	31.323329	22.405138
C	27.206810	30.576195	22.665833
C	33.167036	27.428515	19.285472
C	34.631051	27.185374	18.795620
O	34.621816	26.119751	17.849172
C	35.307980	28.441230	18.105191
O	34.750185	28.584712	16.779779
C	35.497910	28.444307	15.639342
O	35.092032	28.976908	14.630190
C	36.801672	27.696469	15.627047
C	37.919760	28.415667	15.185131
C	39.148908	27.780429	15.050107
C	39.270216	26.415214	15.303202
C	38.150396	25.684349	15.698610
C	36.919972	26.321308	15.871586
C	35.220861	29.816511	18.879850
C	34.384947	30.935118	18.201273
O	32.969875	30.636946	18.399693
C	32.397707	30.979803	19.570619
C	30.903353	30.900354	19.474525
O	33.016458	31.357376	20.548941
C	34.566195	31.391518	16.736143
O	34.944343	32.722369	17.142376
C	34.693489	32.429311	18.536587
C	35.858124	32.790049	19.441530
C	36.398125	31.615318	20.251428
O	37.564384	32.004167	20.955324
C	36.624474	30.331796	19.355685
C	37.549511	30.665634	18.170913
C	37.354080	29.327912	20.288306
O	38.548235	29.170287	20.184707
C	35.531139	26.680768	19.975587
C	35.022279	25.304874	20.509342
C	36.976953	26.363437	19.514478
H	38.280603	25.759221	24.433615
H	39.088229	25.980270	22.844328
H	39.467801	27.053699	24.196390
H	36.341814	29.333716	22.194163
H	34.744858	29.038320	23.369521
H	33.446168	29.874619	22.453571
H	33.107119	28.410911	23.367207
H	32.736911	26.454227	21.194195
H	29.573551	29.290201	21.183924
H	30.682993	30.632147	22.532078
H	28.305898	27.334934	22.349840
H	28.908508	29.036431	24.660706
H	29.373430	28.741337	26.633691
H	29.043310	28.995140	29.064905
H	27.098759	27.940985	30.178205
H	25.450503	26.650918	28.844426
H	25.776523	26.424716	26.372780
H	26.124702	27.536344	21.603844

H	24.100080	28.867703	21.102082
H	24.041873	31.297773	21.639794
H	26.041868	32.385880	22.633160
H	28.077781	31.061868	23.094893
H	32.788332	28.362357	18.866063
H	32.550864	26.629702	18.858286
H	34.019429	26.369899	17.101990
H	36.356562	28.184556	17.970550
H	37.821788	29.471594	14.952997
H	40.008224	28.356934	14.735137
H	40.235909	25.934649	15.187880
H	38.231649	24.617159	15.880060
H	36.059694	25.758192	16.207868
H	34.662302	29.624162	19.789520
H	30.530964	31.671029	18.795067
H	30.583926	29.938696	19.072157
H	30.450579	31.061615	20.450158
H	33.630406	31.370852	16.173068
H	35.345379	30.921903	16.140227
H	33.797962	32.949860	18.878794
H	36.655266	33.201342	18.816193
H	35.573526	33.579686	20.143209
H	35.660480	31.343676	21.011913
H	38.061146	32.629208	20.391521
H	38.329502	31.367012	18.461915
H	38.046697	29.778724	17.771712
H	36.989454	31.147460	17.371049
H	33.947358	25.150612	20.416774
H	35.296056	25.197676	21.561725
H	35.502380	24.501849	19.952772
H	37.513837	25.850060	20.313848
H	36.920034	25.696895	18.652422
H	37.589660	27.214579	19.230201

=== 2* (quartet) ===

h	38.874283	39.025003	22.495440
C	38.654463	38.873825	21.417984
H	37.608780	38.590789	21.289260
H	38.820191	39.830890	20.906453
S	39.780745	37.707293	20.609210
N	40.556266	35.525207	18.837134
C	40.888846	35.947150	17.574237
C	41.736288	35.114738	19.428368
C	42.308618	35.803215	17.356678
C	42.841147	35.306070	18.502392
C	41.780576	34.570630	20.704545
H	42.688772	34.072243	21.014796
C	40.752690	34.589565	21.629348
N	39.485637	35.123820	21.431658
C	38.844106	35.063827	22.665524
C	39.733639	34.487144	23.637831
C	40.892514	34.178942	22.999053
C	37.541700	35.463304	22.900183
H	37.174366	35.390932	23.918778
C	36.666614	35.981625	21.951013
C	35.344687	36.491102	22.235604
C	35.881513	36.833579	20.073249
N	36.969774	36.181681	20.621155
C	34.870135	37.025158	21.083357
C	35.775334	37.276105	18.764329
H	34.849918	37.779296	18.506264
C	36.752117	37.161219	17.781806
C	36.641261	37.653803	16.423796
N	37.998043	36.615637	17.989916
C	38.674792	36.755673	16.801607
C	40.019715	36.453769	16.627876
H	40.467329	36.645989	15.661037
Fe	38.689693	35.695213	19.678812
C	37.831549	37.394660	15.809815
h	38.163808	37.609882	14.872165
h	43.837572	35.153573	18.611882
h	35.738081	38.112733	16.012826
h	42.858397	36.274194	16.534317
h	41.774999	33.679833	23.404417
h	33.938536	37.572204	20.946459
h	34.824488	36.427539	23.193443
h	39.568412	34.515369	24.712971
O	38.176754	34.086093	19.202464
C	38.679718	26.407519	23.747963
C	37.570144	27.347912	23.361757
O	36.700335	27.747700	24.109654
O	37.686134	27.685351	22.065759
C	36.701434	28.570789	21.496684
C	35.462071	27.809911	21.082692
C	34.234897	28.111619	21.565171
C	33.931939	28.955178	22.771184
C	33.029108	27.562368	20.809101
O	31.838171	28.364125	21.073680
C	30.975670	27.956620	22.041403
O	31.131623	26.978027	22.736607
C	29.846248	28.991557	22.175295
O	30.343188	29.994472	23.075248
C	28.518034	28.430353	22.717327
N	28.596232	28.288488	24.169345
C	27.688358	27.484744	24.810795
O	27.008392	26.659638	24.205061
C	27.584665	27.649672	26.299047
C	28.507958	28.362530	27.074778
C	28.317030	28.489076	28.449215
C	27.209464	27.900507	29.059282
C	26.291574	27.180270	28.294122
C	26.482201	27.054586	26.921936
C	27.306189	29.286113	22.336409
C	26.181814	28.679386	21.764503
C	25.030460	29.421646	21.493878
C	24.984181	30.781226	21.802987
C	26.098635	31.396123	22.377732
C	27.249751	30.654985	22.644591
C	33.242115	27.599291	19.278487
C	34.699992	27.316094	18.795584
O	34.656017	26.271332	17.826255
C	35.425416	28.569645	18.137969
O	34.887285	28.757309	16.812466
C	35.624867	28.598882	15.667072
O	35.232484	29.164119	14.669370
C	36.892922	27.792422	15.618224
C	38.000817	28.440013	15.054588
C	39.188482	27.746621	14.854314
C	39.279190	26.391560	15.168064
C	38.168420	25.730957	15.690011
C	36.980307	26.426586	15.923135
C	35.376526	29.934417	18.945418
C	34.603428	31.115045	18.290204
O	33.178352	30.858098	18.429276
C	32.574145	31.161528	19.594667
C	31.082683	31.047906	19.462350
O	33.167342	31.523401	20.593877
C	34.864154	31.630282	16.857860
O	35.424361	32.862842	17.353743
C	34.991111	32.583948	18.705922
C	36.010437	32.832126	19.782472
C	36.635112	31.604554	20.411742
O	37.833422	31.924970	21.093130
C	36.798892	30.374331	19.432713
C	37.730356	30.698933	18.239537
C	37.480568	29.307262	20.343632
O	38.664049	29.088291	20.245382
C	35.568290	26.747115	19.969825
C	34.999288	25.377992	20.468116
C	37.005624	26.385797	19.515035
H	38.288006	25.699443	24.476102
H	39.085116	25.890728	22.877328
H	39.496451	26.969435	24.213713
H	36.447878	29.327420	22.243835
H	34.819217	29.109062	23.382401
H	33.480054	29.913237	22.492375
H	33.200304	28.433692	23.396311
H	32.786268	26.556770	21.147113
H	29.650729	29.416264	21.187615
H	30.630001	30.782232	22.574446
H	28.376502	27.425301	22.311885
H	28.948911	29.109088	24.642206
H	29.392203	28.800565	26.621475
H	29.028994	29.045646	29.049000

```
H     27.066594   27.992640   30.131386
H     25.434587   26.711077   28.770555
H     25.794401   26.492319   26.302553
H     26.201238   27.616162   21.548122
H     24.171724   28.935798   21.037419
H     24.090301   31.361529   21.591720
H     26.071243   32.456409   22.614414
H     28.111228   31.144020   23.089547
H     32.907720   28.565363   18.896365
H     32.595507   26.847677   18.811549
H     34.032960   26.546595   17.103758
H     36.468824   28.287070   18.009024
H     37.922741   29.485240   14.773076
H     40.039148   28.268028   14.437043
H     40.211492   25.863530   14.997028
H     38.222593   24.671597   15.920092
H     36.126430   25.916868   16.347539
H     34.797420   29.744414   19.843641
H     30.700681   31.823353   18.793717
H     30.798448   30.087031   19.030552
H     30.605696   31.167474   20.432312
H     33.929246   31.778449   16.306287
H     35.562989   31.100199   16.215349
H     34.095038   33.170355   18.929802
H     37.113015   33.649201   19.311191
H     35.664119   33.523450   20.551902
H     35.938500   31.270103   21.190470
H     38.341751   32.514259   20.498194
H     38.243200   31.654102   18.370448
H     38.487700   29.924268   18.107746
H     37.172187   30.781124   17.309297
H     33.940711   25.226525   20.256073
H     35.157128   25.281906   21.544666
H     35.533115   24.563964   19.980742
H     37.523418   25.859319   20.318462
H     36.930730   25.717482   18.656045
H     37.644872   27.215645   19.227595

=== 3 (quartet) ===
h     38.970606   39.173151   22.338926
C     38.793709   39.135772   21.242467
H     37.742384   38.918376   21.053306
H     39.022024   40.124757   20.831305
S     39.890055   37.980158   20.354333
N     40.747133   35.762826   18.935472
C     41.079497   36.117132   17.652098
C     41.901957   35.272513   19.527105
C     42.483059   35.889805   17.433456
C     43.000212   35.391151   18.587362
C     41.912346   34.669290   20.773193
H     42.807845   34.143551   21.075591
C     40.856691   34.650608   21.670691
N     39.614439   35.209369   21.475139
C     38.952749   35.138117   22.679580
C     39.797870   34.485135   23.651687
C     40.961572   34.174308   23.027804
C     37.671625   35.608381   22.921787
H     37.276303   35.491493   23.924952
C     36.846497   36.217104   21.989009
C     35.497367   36.665408   22.232579
C     36.092000   37.095615   20.097720
N     37.192783   36.497175   20.679252
C     35.042927   37.213616   21.076409
C     35.979378   37.491694   18.772708
H     35.052397   37.980759   18.495289
C     36.945866   37.319059   17.793946
C     36.838953   37.754064   16.415150
N     38.186062   36.752629   18.013485
C     38.877074   36.872059   16.824844
C     40.221701   36.581709   16.671443
H     40.679879   36.740812   15.703627
Fe    38.925943   36.021177   19.782369
C     38.029418   37.472638   15.813028
h     38.360854   37.645305   14.866344
h     43.986419   35.188089   18.700872
h     35.927347   38.175347   15.981369
h     43.029518   36.293054   16.575643
h     41.822851   33.637065   23.429829
h     34.058121   37.639213   20.888139
h     34.930700   36.515983   23.155201
h     39.610827   34.492503   24.723423
O     38.304864   34.352233   19.353216
C     38.675330   26.384027   23.709846
C     37.555320   27.319213   23.340882
O     36.688810   27.704425   24.100207
O     37.653296   27.668633   22.045918
C     36.640156   28.532782   21.494444
C     35.416313   27.744004   21.086033
C     34.185861   28.024816   21.573051
C     33.880583   28.868732   22.780243
C     32.984092   27.456529   20.824181
O     31.788302   28.255296   21.077738
C     30.926272   27.856537   22.051448
O     31.086983   26.888105   22.759276
C     29.796681   28.893008   22.168318
O     30.287023   29.924674   23.035884
C     28.471797   28.351906   22.733459
N     28.560194   28.224649   24.186163
C     27.657322   27.423078   24.838369
O     26.974507   26.593954   24.241496
C     27.565043   27.594734   26.326140
C     28.498136   28.306118   27.091347
C     28.319680   28.437668   28.466904
C     27.215214   27.855224   29.088302
C     26.287550   27.136491   28.333548
C     26.465425   27.006236   26.960165
C     27.264988   29.214711   22.350637
C     26.133410   28.612655   21.787935
C     24.987883   29.362922   21.513873
C     24.954578   30.725397   21.811810
C     26.076478   31.335416   22.377244
C     27.222453   30.587065   22.646043
C     33.191314   27.462195   19.291499
C     34.655119   27.221326   18.803694
O     34.637468   26.178592   17.831672
C     35.345147   28.491003   18.145122
O     34.803684   28.663725   16.817886
C     35.554021   28.535536   15.677326
O     35.155674   29.097916   14.680577
C     36.843721   27.764691   15.638249
C     37.950379   28.452793   15.122344
C     39.159408   27.792416   14.938590
C     39.273114   26.432068   15.220896
C     38.164469   25.732088   15.694350
C     36.954412   26.394026   15.911728
C     35.253212   29.853724   18.947128
C     34.421343   30.988298   18.279892
O     33.007005   30.676261   18.422255
C     32.401077   30.986495   19.587617
C     30.910091   30.917404   19.449966
O     32.996058   31.330480   20.591981
C     34.674883   31.516302   16.849555
O     35.169751   32.779484   17.349655
C     34.729757   32.452416   18.709819
C     35.718254   32.695699   19.764254
C     36.471727   31.591118   20.404472
O     37.681965   32.046194   20.965907
C     36.663522   30.343096   19.433786
C     37.591580   30.713009   18.255653
C     37.387409   29.309152   20.344845
O     38.582552   29.157312   20.255098
C     35.543785   26.681409   19.975643
C     35.017134   25.301107   20.481192
C     36.989038   26.359404   19.515976
H     38.292187   25.664792   24.431904
H     39.079940   25.879510   22.831602
H     39.489971   26.946074   24.178952
H     36.372176   29.272938   22.253172
H     34.760374   28.991014   23.409803
H     33.466395   29.843540   22.500298
H     33.121765   28.366662   23.387698
H     32.745917   26.456267   21.180028
H     29.590176   29.287324   21.170317
H     30.707626   30.631834   22.511061
```

```
H    28.317263   27.343318   22.341937
H    28.908605   29.051304   24.651661
H    29.380577   28.737986   26.628733
H    29.039074   28.993171   29.058714
H    27.082311   27.951028   30.161283
H    25.432963   26.671993   28.818834
H    25.769883   26.444782   26.348796
H    26.142625   27.547390   21.580556
H    24.123384   28.881365   21.063661
H    24.065245   31.311927   21.598403
H    26.060020   32.398275   22.603819
H    28.091580   31.072239   23.079429
H    32.818150   28.403917   18.885481
H    32.572730   26.674109   18.847710
H    34.012268   26.443236   17.107485
H    36.395856   28.236924   18.017558
H    37.856354   29.503264   14.865931
H    40.008922   28.343992   14.559520
H    40.222764   25.930785   15.065540
H    38.237703   24.668705   15.900073
H    36.102285   25.854499   16.301854
H    34.684754   29.649278   19.848583
H    30.558945   31.707063   18.780954
H    30.596778   29.968223   19.014496
H    30.432444   31.055672   20.417305
H    33.743365   31.623559   16.284743
H    35.406555   31.017477   16.219276
H    33.818804   33.015371   18.932979
H    37.720354   34.394377   18.576816
H    35.815254   33.687460   20.192759
H    35.868884   31.216512   21.244796
H    38.032986   32.761111   20.387070
H    38.173957   31.606381   18.483780
H    38.298024   29.911247   18.031719
H    37.024907   30.937217   17.354929
H    33.944835   25.151209   20.355518
H    35.260441   25.181561   21.539690
H    35.511213   24.502101   19.931333
H    37.516864   25.826542   20.308523
H    36.931330   25.708399   18.642293
H    37.611122   27.209935   19.249602

=== 4* (quartet) ===
h    38.838922   39.113207   22.316549
C    38.630862   39.034365   21.227554
H    37.572399   38.812551   21.080684
H    38.841857   40.008154   20.773525
S    39.696633   37.831312   20.361843
N    40.461649   35.525817   18.882257
C    40.788007   35.889269   17.597600
C    41.642784   35.108060   19.485300
C    42.199130   35.713172   17.381662
C    42.737420   35.263008   18.547295
C    41.682400   34.535139   20.745345
H    42.599672   34.052676   21.054186
C    40.633618   34.496452   21.650936
N    39.371713   35.010269   21.453465
C    38.745552   34.993970   22.680931
C    39.623649   34.396857   23.661029
C    40.778445   34.079892   23.023563
C    37.480755   35.496595   22.942504
H    37.115395   35.424633   23.961496
C    36.646963   36.114296   22.019897
C    35.326798   36.625715   22.296552
C    35.877415   36.999349   20.140841
N    36.962523   36.360080   20.699253
C    34.862719   37.177574   21.146319
C    35.754904   37.396564   18.815894
H    34.845632   37.929324   18.560234
C    36.695251   37.193921   17.817012
C    36.591282   37.677033   16.454413
N    37.911277   36.563887   17.999654
C    38.588357   36.686866   16.804087
C    39.922787   36.361956   16.627692
H    40.373044   36.533003   15.658282
Fe   38.660911   35.830481   19.758070
C    37.756621   37.357051   15.823499
h    38.085312   37.553336   14.880488
h    43.732820   35.110141   18.667335
h    35.703477   38.171787   16.052016
h    42.725249   36.123240   16.513504
h    41.662347   33.582904   23.428755
h    33.906425   37.673073   20.990944
h    34.786557   36.523528   23.239407
h    39.470541   34.457730   24.736751
O    37.785970   34.076732   19.384597
C    38.699653   26.327781   23.759939
C    37.590153   27.265336   23.371679
O    36.733339   27.689317   24.120810
O    37.689527   27.573890   22.065763
C    36.724990   28.483629   21.505484
C    35.470047   27.757942   21.080271
C    34.249004   28.081929   21.563234
C    33.957886   28.923007   22.773819
C    33.034737   27.559488   20.801521
O    31.855745   28.374279   21.071834
C    30.989276   27.970435   22.038338
O    31.139488   26.988806   22.730174
C    29.865896   29.011828   22.171185
O    30.372264   30.013326   23.067902
C    28.535889   28.455122   22.712892
N    28.613992   28.309685   24.164429
C    27.708633   27.498404   24.801237
O    27.034834   26.672180   24.190331
C    27.600574   27.657063   26.289463
C    28.521868   28.366482   27.070754
C    28.326686   28.487413   28.445074
C    27.217103   27.896633   29.049303
C    26.301360   27.179680   28.278543
C    26.496133   27.059430   26.906477
C    27.325538   29.312727   22.332333
C    26.201263   28.706830   21.759143
C    25.050009   29.449403   21.489314
C    25.003787   30.808581   21.800151
C    26.118160   31.422767   22.375805
C    27.269245   30.681234   22.642026
C    33.248002   27.609814   19.271525
C    34.700461   27.309379   18.786555
O    34.641848   26.280407   17.801424
C    35.446475   28.561652   18.147428
O    34.915112   28.780296   16.824545
C    35.650118   28.633845   15.674091
O    35.261420   29.225588   14.690065
C    36.907214   27.813669   15.603802
C    38.001899   28.442530   14.993757
C    39.180174   27.739355   14.775711
C    39.274581   26.391615   15.119166
C    38.176053   25.748421   15.686531
C    36.997463   26.454486   15.936263
C    35.420093   29.916120   18.974884
C    34.677355   31.124542   18.328768
O    33.249450   30.891490   18.425718
C    32.626842   31.181662   19.587937
C    31.137615   31.070841   19.437444
O    33.209913   31.531435   20.597142
C    35.004402   31.677877   16.925324
O    35.635148   32.853581   17.491613
C    35.086924   32.565602   18.808152
C    36.031943   32.719756   19.940746
C    36.731748   31.520754   20.481314
O    37.953449   31.874159   21.082604
C    36.854273   30.309900   19.467745
C    37.787981   30.627898   18.274817
C    37.517120   29.215943   20.361818
O    38.697606   28.984441   20.263131
C    35.555756   26.707736   19.954470
C    34.956569   25.344657   20.436585
C    36.986279   26.324656   19.496929
H    38.313333   25.630909   24.500951
H    39.095743   25.799249   22.892329
H    39.521517   26.894101   24.210885
H    36.485763   29.234090   22.263399
H    34.848362   29.081193   23.379457
H    33.498887   29.879104   22.500172
```

H	33.232322	28.398783	23.403776
H	32.780078	26.552917	21.128698
H	29.673136	29.435360	21.182193
H	30.627111	30.812668	22.568324
H	28.391953	27.450750	22.306582
H	28.958348	29.131834	24.640814
H	29.407521	28.806225	26.621799
H	29.036665	29.041443	29.049390
H	27.070907	27.984545	30.121278
H	25.442845	26.708904	28.750561
H	25.809993	26.499802	26.282852
H	26.220714	27.643902	21.541276
H	24.191326	28.964137	21.032215
H	24.109898	31.389087	21.589619
H	26.090207	32.482552	22.614257
H	28.130155	31.170183	23.088443
H	32.928398	28.584796	18.899296
H	32.589018	26.874067	18.796619
H	34.012714	26.571292	17.089867
H	36.485603	28.264776	18.016922
H	37.919515	29.480821	14.689312
H	40.020343	28.246992	14.321891
H	40.199681	25.855588	14.934813
H	38.232362	24.694451	15.939543
H	36.151108	25.958360	16.390914
H	34.830210	29.729334	19.867184
H	30.760503	31.863813	18.786668
H	30.857501	30.121459	18.978206
H	30.653079	31.164560	20.406427
H	34.093749	31.920892	16.367184
H	35.678340	31.132864	16.270202
H	34.208531	33.194213	18.978559
H	37.517356	34.056123	18.447772
H	35.838384	33.505277	20.658653
H	36.085907	31.153459	21.293394
H	38.312324	32.589561	20.513979
H	38.247010	31.612405	18.376250
H	38.584035	29.886804	18.194286
H	37.248512	30.628835	17.329875
H	33.908036	25.199286	20.175099
H	35.064878	25.253524	21.519205
H	35.507548	24.523800	19.981082
H	37.498751	25.793598	20.300462
H	36.899143	25.655204	18.640344
H	37.636550	27.144158	19.203867

=== 5 (quartet) ===

h	39.023082	39.446762	22.456130
C	38.889364	39.451947	21.353720
H	37.850016	39.207065	21.122028
H	39.089365	40.467509	20.996765
S	40.045333	38.340120	20.480258
N	40.732798	35.899765	18.880611
C	41.085086	36.293349	17.609817
C	41.894349	35.417044	19.473918
C	42.492117	36.092351	17.405050
C	43.000489	35.568501	18.552931
C	41.899511	34.794037	20.710592
H	42.797669	34.276591	21.018661
C	40.836666	34.750765	21.596918
N	39.584890	35.296165	21.387464
C	38.924520	35.197424	22.595916
C	39.765157	34.535300	23.564087
C	40.939012	34.252911	22.945734
C	37.650043	35.676711	22.848701
H	37.254152	35.550602	23.850888
C	36.837813	36.317127	21.927209
C	35.493741	36.768967	22.182133
C	36.090180	37.229708	20.055314
N	37.191928	36.612864	20.621047
C	35.041985	37.339102	21.035428
C	35.978122	37.630241	18.732338
H	35.051432	38.119487	18.452471
C	36.947553	37.458126	17.755430
C	36.825046	37.866964	16.370453
N	38.200940	36.923699	17.986465
C	38.878459	37.030399	16.786719
C	40.225927	36.755681	16.629537
H	40.682809	36.925171	15.662788
Fe	38.993150	36.367353	19.784489
C	38.017545	37.595692	15.767393
h	38.344361	37.762970	14.818380
h	43.987078	35.366239	18.664559
h	35.899241	38.249605	15.931354
h	43.056639	36.513236	16.567599
h	41.821641	33.761903	23.360577
h	34.057548	37.770277	20.861694
h	34.929407	36.610207	23.105504
h	39.570582	34.523304	24.634756
O	37.076219	33.429794	19.274289
C	38.715243	26.354308	23.804846
C	37.568328	27.241172	23.406959
O	36.733794	27.695193	24.163156
O	37.600752	27.464785	22.079644
C	36.629490	28.365793	21.517794
C	35.384427	27.632436	21.079526
C	34.159981	27.946305	21.559799
C	33.870341	28.783232	22.775870
C	32.948252	27.423329	20.793785
O	31.773862	28.250574	21.050926
C	30.907757	27.867601	22.028599
O	31.053995	26.892766	22.730379
C	29.798429	28.924600	22.158199
O	30.314894	29.936380	23.035795
C	28.468168	28.393442	22.721133
N	28.560678	28.249529	24.171977
C	27.660742	27.437303	24.816317
O	26.983215	26.610238	24.210787
C	27.565602	27.595523	26.305100
C	28.489399	28.311858	27.077108
C	28.307614	28.430319	28.453324
C	27.209540	27.829846	29.068751
C	26.291063	27.106615	28.307179
C	26.472024	26.989384	26.933031
C	27.266605	29.269056	22.353578
C	26.124249	28.677443	21.801367
C	24.979874	29.436170	21.547502
C	24.959144	30.797236	21.852943
C	26.092295	31.397270	22.406609
C	27.236484	30.639813	22.657477
C	33.163726	27.445084	19.261758
C	34.621712	27.166788	18.777976
O	34.587373	26.134619	17.795916
C	35.343102	28.424594	18.134578
O	34.796083	28.648545	16.817474
C	35.538110	28.539748	15.666187
O	35.135072	29.127421	14.686322
C	36.828330	27.774069	15.610508
C	37.921321	28.469003	15.074538
C	39.140712	27.826123	14.901700
C	39.277147	26.474044	15.212799
C	38.181007	25.765022	15.702625
C	36.960989	26.411350	15.911725
C	35.305382	29.763801	18.970517
C	34.549293	30.965049	18.335419
O	33.120265	30.724744	18.434793
C	32.504776	31.022499	19.599829
C	31.015162	30.963021	19.447532
O	33.097417	31.348730	20.612027
C	34.852005	31.511208	16.921548
O	35.322454	32.774497	17.461972
C	34.904089	32.416765	18.797702
C	36.008663	32.677050	19.841368
C	36.538026	31.381272	20.470550
O	37.692691	31.669702	21.226635
C	36.729150	30.176091	19.470675
C	37.680844	30.487258	18.284730
C	37.414673	29.118029	20.390440
O	38.610380	28.957399	20.330122
C	35.490263	26.590023	19.949413
C	34.927598	25.216397	20.434999
C	36.929834	26.243257	19.492281
H	38.358408	25.646170	24.550474
H	39.136433	25.836557	22.942935

H	39.507316	26.959652	24.258247
H	36.381082	29.112364	22.276686
H	34.755003	28.909029	23.398097
H	33.448558	29.757623	22.506290
H	33.118544	28.276929	23.388387
H	32.683537	26.423841	21.133791
H	29.597400	29.334795	21.165115
H	30.678156	30.676858	22.514999
H	28.302389	27.390047	22.320989
H	28.902314	29.074231	24.645893
H	29.367676	28.757876	26.619942
H	29.019259	28.990054	29.050280
H	27.074539	27.915575	30.142290
H	25.441239	26.628657	28.787685
H	25.783343	26.425276	26.316335
H	26.124350	27.613541	21.586975
H	24.106611	28.962028	21.106696
H	24.070510	31.389964	21.654463
H	26.085396	32.458826	22.639230
H	28.113307	31.117400	23.084239
H	32.822044	28.404624	18.868608
H	32.522451	26.684444	18.802831
H	33.976840	26.419861	17.066262
H	36.383491	28.140024	17.991720
H	37.809438	29.512414	14.796867
H	39.979614	28.386073	14.511350
H	40.235425	25.986595	15.067871
H	38.272690	24.707604	15.930549
H	36.117689	25.867629	16.316315
H	34.719569	29.562426	19.860953
H	30.673608	31.753675	18.774934
H	30.701509	30.014165	19.010857
H	30.530079	31.101601	20.411045
H	33.948886	31.634411	16.318846
H	35.617271	31.030767	16.318671
H	34.014167	32.982818	19.072775
H	36.908315	33.452576	18.313574
H	35.597007	33.274753	20.659887
H	35.776892	31.057895	21.183785
H	38.263847	32.213078	20.653106
H	38.064541	31.506470	18.324733
H	38.533959	29.808091	18.284557
H	37.178161	30.374239	17.324853
H	33.859525	25.080891	20.264162
H	35.127383	25.095147	21.501877
H	35.435885	24.411023	19.909165
H	37.452820	25.715883	20.291071
H	36.861866	25.583577	18.626365
H	37.561593	27.082177	19.211082

=== 1 (sextet) ===

h	39.058407	39.196379	22.587591
C	38.895010	39.107956	21.494004
H	37.859941	38.827059	21.297093
H	39.084310	40.094446	21.049830
S	40.055871	37.991355	20.670898
N	40.917923	35.829751	18.897779
C	41.262970	36.257466	17.641534
C	42.053415	35.353648	19.512884
C	42.682603	36.053709	17.450291
C	43.172628	35.513732	18.600952
C	42.041329	34.800145	20.796831
H	42.926855	34.264723	21.113648
C	41.009478	34.835375	21.726368
N	39.760396	35.402667	21.529183
C	39.060450	35.299229	22.719277
C	39.909527	34.654668	23.693310
C	41.091571	34.363598	23.085391
C	37.740500	35.701054	22.907731
H	37.332559	35.571818	23.905466
C	36.871585	36.242011	21.954612
C	35.503291	36.663562	22.183665
C	36.106441	37.064521	20.036893
N	37.206438	36.501049	20.647672
C	35.041657	37.174796	21.011909
C	36.035568	37.463846	18.702575
H	35.089301	37.901163	18.400119
C	37.037575	37.377657	17.727016
C	36.942625	37.819390	16.343950
N	38.306766	36.916502	17.967962
C	39.024922	37.057530	16.807681
C	40.389038	36.777310	16.689222
H	40.847147	36.973573	15.728652
Fe	38.956755	35.896374	19.675264
C	38.169458	37.621122	15.774500
h	38.516444	37.816133	14.838041
h	44.161340	35.331583	18.724027
h	36.019413	38.186074	15.886437
h	43.297711	36.519415	16.672992
h	41.949330	33.829749	23.498268
h	34.061401	37.609804	20.822728
h	34.944638	36.530719	23.114160
h	39.699543	34.627091	24.759347
O	38.465797	34.405766	19.267386
C	38.670886	26.484783	23.715533
C	37.543249	27.404534	23.328176
O	36.677665	27.800077	24.083381
O	37.632389	27.723097	22.024945
C	36.609176	28.564504	21.454592
C	35.391498	27.753360	21.066013
C	34.160670	28.030626	21.554700
C	33.856928	28.893497	22.749272
C	32.959532	27.438471	20.822855
O	31.758517	28.232517	21.073579
C	30.907885	27.841861	22.060599
O	31.077037	26.879966	22.775454
C	29.776333	28.875955	22.185374
O	30.266383	29.903195	23.059145
C	28.453842	28.328548	22.751878
N	28.546345	28.199938	24.204206
C	27.644265	27.401293	24.860276
O	26.956933	26.573260	24.266953
C	27.558363	27.574117	26.348544
C	28.490842	28.291825	27.108564
C	28.318862	28.423334	28.484953
C	27.220840	27.835112	29.112353
C	26.293510	27.110526	28.362759
C	26.465367	26.979932	26.988623
C	27.243127	29.188665	22.375247
C	26.110137	28.584390	21.817899
C	24.961013	29.331870	21.551451
C	24.925586	30.693819	21.851534
C	26.048879	31.306061	22.411746
C	27.198174	30.560330	22.673352
C	33.156868	27.417149	19.288013
C	34.620291	27.176852	18.795239
O	34.611949	26.112496	17.847537
C	35.293153	28.434848	18.105106
O	34.733546	28.579017	16.780652
C	35.481594	28.442686	15.639732
O	35.074321	28.975759	14.631492
C	36.787692	27.699149	15.627784
C	37.905405	28.424035	15.194402
C	39.137397	27.793541	15.062932
C	39.261846	26.427631	15.310682
C	38.142459	25.691189	15.697093
C	36.909135	26.323353	15.866919
C	35.203695	29.808125	18.882033
C	34.366372	30.928376	18.207130
O	32.951912	30.628785	18.405089
C	32.379762	30.970281	19.576529
C	30.885481	30.889231	19.481365
O	32.998744	31.347749	20.554749
C	34.549423	31.389561	16.743689
O	34.937791	32.715919	17.155554
C	34.675898	32.421528	18.547479
C	35.834137	32.779563	19.462391
C	36.374293	31.599101	20.263631
O	37.536247	31.981943	20.978813
C	36.606219	30.324474	19.358995
C	37.527925	30.668513	18.174672
C	37.342865	29.320326	20.285873
O	38.537493	29.168586	20.177751
C	35.523274	26.672349	19.973288

C	35.016900	25.295513	20.506988
C	36.968575	26.357417	19.509158
H	38.287076	25.760466	24.433177
H	39.095402	25.983968	22.844631
H	39.469358	27.059385	24.197173
H	36.336271	29.323221	22.193147
H	34.740767	29.028889	23.370103
H	33.439391	29.863044	22.456109
H	33.103846	28.399118	23.370216
H	32.731089	26.443180	21.197661
H	29.566108	29.276713	21.191074
H	30.673823	30.620855	22.537998
H	28.301311	27.320371	22.358442
H	28.901724	29.024537	24.667957
H	29.368651	28.728115	26.641396
H	29.038566	28.982978	29.072694
H	27.092874	27.930970	30.185961
H	25.443845	26.641639	28.852529
H	25.770177	26.413889	26.381092
H	26.121089	27.519426	21.609249
H	24.095364	28.848453	21.105477
H	24.033469	31.278195	21.643980
H	26.030858	32.368497	22.640164
H	28.068027	31.047021	23.103554
H	32.775299	28.350024	18.868904
H	32.541486	26.616875	18.862486
H	34.009276	26.362800	17.100639
H	36.342163	28.180792	17.969019
H	37.805222	29.480691	14.966465
H	39.996353	28.374504	14.755174
H	40.229757	25.950903	15.198338
H	38.226313	24.623453	15.874198
H	36.049073	25.756026	16.196745
H	34.645633	29.612874	19.791331
H	30.511636	31.659859	18.802689
H	30.566853	29.927448	19.078700
H	30.433161	31.049709	20.457321
H	33.612299	31.378799	16.182389
H	35.324107	30.917300	16.144122
H	33.778605	32.943419	18.882987
H	36.632216	33.198773	18.843269
H	35.542992	33.562006	20.169437
H	35.634958	31.320475	21.019428
H	38.056518	32.578785	20.407224
H	38.318937	31.354951	18.471117
H	38.012313	29.782565	17.757969
H	36.968970	31.170717	17.386688
H	33.942094	25.139843	20.415262
H	35.291695	25.188278	21.559103
H	35.497784	24.493471	19.949757
H	37.508217	25.845435	20.307561
H	36.910988	25.690604	18.647383
H	37.579017	27.209632	19.222989

=== 2* (sextet) ===

h	39.014287	39.237030	22.683459
C	38.847972	39.102270	21.596491
H	37.812498	38.808276	21.409047
H	39.018296	40.070704	21.105169
S	40.009672	37.959463	20.817301
N	40.642651	35.624932	18.811269
C	40.974915	36.066050	17.554440
C	41.814214	35.225067	19.430964
C	42.400773	35.947367	17.367786
C	42.924026	35.442830	18.519308
C	41.839652	34.685141	20.716332
H	42.749703	34.196858	21.037556
C	40.809138	34.690623	21.648682
N	39.543442	35.210987	21.448020
C	38.883175	35.151172	22.661745
C	39.763252	34.562970	23.644371
C	40.936689	34.272668	23.021145
C	37.580157	35.590610	22.880663
H	37.201799	35.509352	23.895175
C	36.713376	36.153938	21.942570
C	35.385163	36.660397	22.214446
C	35.935994	37.016701	20.048028
N	37.017790	36.375817	20.616028
C	34.916482	37.197124	21.057076
C	35.842457	37.429941	18.719286
H	34.919384	37.935195	18.453917
C	36.808739	37.293503	17.718558
C	36.716036	37.789171	16.354827
N	38.044816	36.721878	17.913037
C	38.744305	36.864305	16.738177
C	40.096929	36.560446	16.595807
H	40.555433	36.753404	15.633737
Fe	38.664600	35.576555	19.564978
C	37.907872	37.515391	15.745290
h	38.249886	37.719438	14.809114
h	43.920695	35.296297	18.632886
h	35.823110	38.266415	15.942426
h	42.968499	36.424582	16.562149
h	41.829489	33.805606	23.440402
h	33.961273	37.696359	20.905228
h	34.850465	36.565602	23.162651
h	39.581652	34.574206	24.716242
O	38.060951	33.942661	19.232897
C	38.710141	26.411001	23.762950
C	37.584095	27.325423	23.364000
O	36.729860	27.752855	24.114421
O	37.662292	27.603822	22.050176
C	36.676784	28.486370	21.480304
C	35.434436	27.729324	21.069334
C	34.210540	28.040822	21.553522
C	33.919673	28.896793	22.755169
C	32.998472	27.495286	20.804155
O	31.815338	28.310209	21.064788
C	30.956106	27.921604	22.044685
O	31.110596	26.949940	22.749543
C	29.835111	28.966426	22.173739
O	30.336999	29.977881	23.060871
C	28.505326	28.419754	22.724769
N	28.585989	28.283120	24.176963
C	27.679097	27.479108	24.820291
O	27.000231	26.652362	24.215614
C	27.576338	27.645025	26.308059
C	28.498653	28.360789	27.082284
C	28.309777	28.485717	28.457057
C	27.205052	27.893380	29.068580
C	26.287765	27.170984	28.304778
C	26.476507	27.046527	26.932318
C	27.298741	29.282253	22.342323
C	26.170219	28.680362	21.773362
C	25.022818	29.428566	21.502257
C	24.984875	30.789218	21.807826
C	26.103865	31.399364	22.378891
C	27.251203	30.652475	22.646152
C	33.204332	27.509054	19.271046
C	34.661133	27.228168	18.782442
O	34.619715	26.182451	17.814978
C	35.378776	28.480415	18.119437
O	34.835592	28.670233	16.796185
C	35.582780	28.539481	15.652085
O	35.190136	29.114149	14.660215
C	36.863017	27.755039	15.609588
C	37.969829	28.428627	15.075430
C	39.174285	27.759256	14.896433
C	39.282159	26.403210	15.200574
C	38.172716	25.716730	15.692004
C	36.967579	26.388741	15.906225
C	35.326021	29.838501	18.928259
C	34.542366	31.016190	18.279248
O	33.118770	30.761754	18.431211
C	32.523505	31.087536	19.596748
C	31.031010	31.002398	19.471299
O	33.126753	31.451918	20.589360
C	34.794717	31.536975	16.846175
O	35.334685	32.779057	17.345364
C	34.918999	32.482275	18.693300
C	35.958700	32.725824	19.758892
C	36.556506	31.494859	20.406549
O	37.726387	31.815447	21.127562
C	36.746976	30.281426	19.417546

C	37.681828	30.625660	18.231425
C	37.445385	29.222312	20.325512
O	38.632321	29.024173	20.224007
C	35.536000	26.666420	19.956485
C	34.973866	25.298842	20.462692
C	36.972339	26.308452	19.496824
H	38.329593	25.702673	24.496926
H	39.128781	25.893275	22.899440
H	39.511832	26.994118	24.228783
H	36.427044	29.244790	22.226831
H	34.807990	29.042562	23.367284
H	33.484688	29.860911	22.470071
H	33.178551	28.392539	23.382725
H	32.748031	26.495857	21.154219
H	29.636961	29.380676	21.182289
H	30.669632	30.738261	22.547251
H	28.354505	27.413671	22.325354
H	28.934937	29.106340	24.648056
H	29.380888	28.801669	26.627836
H	29.021246	29.043693	29.056073
H	27.063991	27.984372	30.140927
H	25.432892	26.699051	28.782309
H	25.789394	26.482390	26.313910
H	26.183346	27.616511	21.559269
H	24.160654	28.946580	21.048208
H	24.094027	31.374047	21.596217
H	26.083349	32.460634	22.611899
H	28.116930	31.137877	23.086667
H	32.860283	28.466508	18.875414
H	32.561723	26.745056	18.819634
H	34.014908	26.464276	17.079599
H	36.422453	28.201597	17.986411
H	37.879412	29.475342	14.803080
H	40.024642	28.301081	14.505354
H	40.228394	25.895228	15.047429
H	38.241920	24.656614	15.914847
H	36.114344	25.861157	16.310718
H	34.749491	29.643132	19.826498
H	30.663576	31.784947	18.802836
H	30.725343	30.047090	19.042818
H	30.559941	31.137309	20.442076
H	33.859918	31.674695	16.293455
H	35.504877	31.020945	16.205525
H	34.027712	33.063299	18.943982
H	36.943920	33.391412	19.261015
H	35.636025	33.438361	20.520955
H	35.826293	31.161070	21.153435
H	38.254141	32.407338	20.551545
H	38.163977	31.597136	18.357602
H	38.466620	29.875680	18.121177
H	37.133738	30.677402	17.292276
H	33.908098	25.155319	20.284483
H	35.165016	25.196426	21.533226
H	35.488356	24.485568	19.954568
H	37.496118	25.787686	20.299880
H	36.896178	25.637601	18.640101
H	37.606773	27.140011	19.202867

=== 3 (sextet) ===

h	39.031818	39.279362	22.661785
C	38.874989	39.167225	21.570646
H	37.840835	38.879538	21.367891
H	39.052997	40.145143	21.101156
S	40.042484	38.037174	20.780894
N	40.710125	35.725161	18.752226
C	41.045527	36.157612	17.494590
C	41.873298	35.307478	19.371633
C	42.471202	36.026234	17.310015
C	42.986917	35.514429	18.461479
C	41.886041	34.749600	20.649869
H	42.790441	34.251103	20.971071
C	40.846664	34.742715	21.573207
N	39.582498	35.265612	21.370257
C	38.911922	35.187540	22.577221
C	39.781140	34.579013	23.556142
C	40.960193	34.298923	22.938526
C	37.609800	35.631640	22.793674
H	37.222830	35.535411	23.803652
C	36.753923	36.217621	21.859323
C	35.424643	36.722167	22.130370
C	35.989835	37.110423	19.971418
N	37.067070	36.461011	20.538430
C	34.964069	37.276832	20.977921
C	35.905906	37.534104	18.644460
H	34.987138	38.045611	18.376302
C	36.875605	37.394368	17.645633
C	36.791122	37.883767	16.279999
N	38.105573	36.806397	17.840870
C	38.810502	36.937166	16.665740
C	40.165376	36.634966	16.528406
H	40.623946	36.818734	15.564632
Fe	38.724369	35.679568	19.494303
C	37.979363	37.587375	15.670312
h	38.321578	37.773461	14.730548
h	43.981125	35.353935	18.575146
h	35.904270	38.370955	15.865265
h	43.047593	36.499281	16.508798
h	41.846146	33.817768	23.356169
h	34.001982	37.762896	20.827612
h	34.882381	36.610535	23.073493
h	39.587379	34.566252	24.625920
O	38.082398	33.989771	19.112884
C	38.778113	26.336179	23.783159
C	37.609224	27.205132	23.408294
O	36.778050	27.638591	24.180933
O	37.619857	27.441210	22.083261
C	36.637557	28.347131	21.546924
C	35.394353	27.616811	21.103896
C	34.170883	27.927369	21.588132
C	33.879195	28.759171	22.806092
C	32.957432	27.416090	20.817195
O	31.783033	28.240287	21.078246
C	30.908935	27.857658	22.046510
O	31.050102	26.888018	22.756908
C	29.795784	28.913712	22.143972
O	30.297368	29.960459	22.985527
C	28.465107	28.399216	22.715661
N	28.549780	28.275109	24.169018
C	27.649785	27.464590	24.814865
O	26.977249	26.631490	24.211772
C	27.546448	27.629636	26.302184
C	28.469475	28.343054	27.077568
C	28.280507	28.464327	28.452632
C	27.176061	27.870444	29.062956
C	26.258211	27.150297	28.297785
C	26.446438	27.029803	26.925048
C	27.267397	29.271852	22.327798
C	26.130501	28.675085	21.769715
C	24.989906	29.432027	21.493993
C	24.967153	30.796268	21.784732
C	26.094996	31.401253	22.343839
C	27.236366	30.646319	22.614713
C	33.171992	27.457569	19.287065
C	34.630853	27.201352	18.800483
O	34.594924	26.198721	17.786760
C	35.351139	28.481634	18.197571
O	34.827476	28.724361	16.873596
C	35.579785	28.626516	15.729646
O	35.191455	29.233362	14.754862
C	36.856125	27.836426	15.654833
C	37.950400	28.508566	15.091826
C	39.147590	27.837434	14.876045
C	39.261196	26.479885	15.172605
C	38.163825	25.794541	15.691234
C	36.966182	26.468267	15.940324
C	35.289870	29.812549	19.055719
C	34.507418	31.004496	18.428985
O	33.081843	30.723156	18.473505
C	32.413280	30.980272	19.618751
C	30.931122	30.945304	19.398063
O	32.951964	31.254735	20.675218
C	34.874680	31.629907	17.065924
O	35.437012	32.802169	17.710356
C	34.831094	32.416378	18.998319

C	35.713910	32.524732	20.157295
C	36.614554	31.424690	20.588170
O	37.867228	31.925016	20.994507
C	36.723773	30.223559	19.546550
C	37.653881	30.559057	18.354178
C	37.415985	29.142693	20.433594
O	38.614156	28.998022	20.382718
C	35.497290	26.594448	19.957114
C	34.928881	25.213707	20.414816
C	36.937750	26.255442	19.496900
H	38.448311	25.620557	24.533400
H	39.192954	25.828478	22.912254
H	39.567441	26.954058	24.224631
H	36.384746	29.064225	22.331107
H	34.761561	28.893176	23.430299
H	33.451565	29.729596	22.533824
H	33.128860	28.247688	23.415667
H	32.691662	26.412498	21.144088
H	29.594597	29.286373	21.135729
H	30.773791	30.618026	22.444254
H	28.297428	27.390106	22.331192
H	28.886626	29.104938	24.637468
H	29.351646	28.784930	26.623843
H	28.991571	29.020925	29.053059
H	27.035569	27.958620	30.135534
H	25.403577	26.677012	28.774391
H	25.759030	26.467247	26.305545
H	26.132158	27.608940	21.566318
H	24.121307	28.954295	21.047707
H	24.081604	31.387915	21.569500
H	26.087126	32.465780	22.562616
H	28.111624	31.127805	23.039799
H	32.820932	28.417263	18.903622
H	32.537940	26.695613	18.819806
H	33.960426	26.501355	17.085060
H	36.396608	28.209391	18.067280
H	37.854503	29.555836	14.824447
H	39.988238	28.378393	14.462762
H	40.201651	25.969798	14.992144
H	38.235944	24.733202	15.908049
H	36.120652	25.940598	16.360145
H	34.707646	29.597494	19.946109
H	30.625502	31.781386	18.764519
H	30.627821	30.031174	18.886935
H	30.404434	31.031578	20.346002
H	33.983350	31.866860	16.475167
H	35.597034	31.135183	16.422852
H	33.928689	33.014309	19.149691
H	37.309795	33.844561	18.537951
H	35.688086	33.411576	20.781011
H	36.177521	30.984359	21.493729
H	38.124188	32.605768	20.327099
H	38.032269	31.580694	18.401877
H	38.512367	29.887134	18.336695
H	37.140287	30.450399	17.400258
H	33.864507	25.078731	20.220674
H	35.107681	25.078131	21.483719
H	35.449664	24.416336	19.888974
H	37.454347	25.704254	20.283855
H	36.873406	25.619459	18.613127
H	37.573700	27.100534	19.243500

=== 4* (sextet) ===

h	38.932017	39.217657	22.601372
C	38.748442	39.074655	21.517526
H	37.704418	38.798089	21.348850
H	38.925074	40.033944	21.012086
S	39.872161	37.890497	20.737740
N	40.514699	35.522229	18.764012
C	40.837096	35.970785	17.507102
C	41.694640	35.151587	19.386915
C	42.266605	35.879976	17.318637
C	42.800816	35.392592	18.472710
C	41.727704	34.608140	20.670914
H	42.651653	34.149581	20.996120
C	40.693964	34.578110	21.604889
N	39.413849	35.052676	21.407718
C	38.777232	35.035686	22.634802
C	39.680767	34.487541	23.620980
C	40.849300	34.199165	22.987414
C	37.486509	35.506923	22.870815
H	37.128192	35.449358	23.894612
C	36.617358	36.098194	21.949089
C	35.307841	36.642548	22.242420
C	35.837015	36.989558	20.068775
N	36.904587	36.318326	20.620617
C	34.837573	37.196511	21.093989
C	35.739862	37.408148	18.739243
H	34.833654	37.951615	18.490383
C	36.683659	37.242779	17.720111
C	36.596271	37.768094	16.368480
N	37.897886	36.611041	17.881318
C	38.595319	36.763412	16.705390
C	39.947548	36.453164	16.552188
H	40.399399	36.655148	15.588601
Fe	38.544635	35.537721	19.561160
C	37.769482	37.460506	15.736708
h	38.106437	37.672071	14.800502
h	43.800917	35.267679	18.584762
h	35.716912	38.283722	15.975016
h	42.824500	36.371862	16.514009
h	41.755762	33.758043	23.405478
h	33.894702	37.722080	20.958713
h	34.785726	36.563202	23.197935
h	39.518434	34.530167	24.695590
O	37.721147	33.815099	19.224769
C	38.749494	26.360444	23.808005
C	37.610141	27.256563	23.409054
O	36.768981	27.704303	24.162036
O	37.661565	27.498695	22.085913
C	36.703162	28.413440	21.524236
C	35.444970	27.700640	21.090149
C	34.225946	28.034905	21.570529
C	33.941220	28.878268	22.781488
C	33.008324	27.526846	20.804205
O	31.836903	28.353408	21.071751
C	30.968827	27.963168	22.042432
O	31.113296	26.985333	22.740876
C	29.852535	29.013071	22.168434
O	30.359748	30.019240	23.058980
C	28.519650	28.466503	22.712234
N	28.595522	28.326373	24.164320
C	27.691034	27.514813	24.801854
O	27.018820	26.686604	24.191819
C	27.582361	27.674909	26.289725
C	28.501095	28.388410	27.070322
C	28.305887	28.508701	28.444632
C	27.198920	27.913663	29.049390
C	26.285603	27.193003	28.279267
C	26.480398	27.073255	26.907236
C	27.313483	29.327555	22.326743
C	26.187583	28.723407	21.754851
C	25.039171	29.469060	21.481517
C	24.997732	30.829613	21.787035
C	26.114147	31.442137	22.360571
C	27.262408	30.697564	22.630600
C	33.223389	27.573702	19.274057
C	34.675217	27.270754	18.789725
O	34.616142	26.248781	17.797315
C	35.424574	28.524053	18.160125
O	34.897481	28.756653	16.837393
C	35.639214	28.628241	15.688513
O	35.254154	29.231588	14.710099
C	36.899780	27.814582	15.615563
C	37.990913	28.451388	15.007102
C	39.174927	27.757279	14.792168
C	39.278018	26.410374	15.136721
C	38.183044	25.758920	15.701919
C	36.998767	26.456429	15.949316
C	35.400504	29.868599	18.999387
C	34.668198	31.088713	18.363270
O	33.237017	30.864405	18.444709
C	32.604080	31.149399	19.603178
C	31.115680	31.062050	19.433040

```
O    33.178214   31.478099   20.624386
C    35.007021   31.655217   16.967641
O    35.613321   32.837911   17.548115
C    35.074313   32.519341   18.869314
C    36.035008   32.659889   19.990396
C    36.713461   31.444346   20.525060
O    37.934562   31.772854   21.147628
C    36.836240   30.246313   19.497988
C    37.775078   30.558890   18.305815
C    37.498202   29.148073   20.387854
O    38.681191   28.925771   20.295347
C    35.526746   26.659687   19.956325
C    34.922732   25.296880   20.430793
C    36.957065   26.275736   19.499686
H    38.387521   25.658199   24.556148
H    39.164915   25.837017   22.946667
H    39.548224   26.960254   24.257333
H    36.466381   29.161440   22.284905
H    34.831175   29.030331   23.389872
H    33.492672   29.838718   22.506126
H    33.208530   28.362059   23.409326
H    32.743033   26.522679   21.130262
H    29.662038   29.430488   21.176363
H    30.652395   30.799192   22.549920
H    28.371031   27.460895   22.310793
H    28.938030   29.150081   24.639290
H    29.384947   28.831635   26.621235
H    29.013896   29.065517   29.048617
H    27.052947   28.001238   30.121362
H    25.429118   26.718880   28.751643
H    25.796351   26.510649   26.284046
H    26.203671   27.659691   21.540295
H    24.179077   28.985112   21.025704
H    24.106058   31.412466   21.573564
H    26.090350   32.503190   22.593831
H    28.125655   31.184992   23.074069
H    32.905374   28.548467   18.899457
H    32.564061   26.837833   18.800083
H    33.995928   26.551079   17.082543
H    36.462846   28.225268   18.029136
H    37.901396   29.489077   14.702750
H    40.013050   28.271572   14.341664
H    40.207907   25.881918   14.955247
H    38.246963   24.705591   15.956006
H    36.154379   25.954964   16.402433
H    34.806417   29.678028   19.887784
H    30.759802   31.860788   18.777660
H    30.826993   30.117150   18.970026
H    30.619422   31.163967   20.395388
H    34.103348   31.890964   16.396431
H    35.699176   31.123128   16.321328
H    34.197525   33.144778   19.053660
H    37.326834   33.699827   18.341132
H    35.850610   33.440955   20.716652
H    36.065244   31.069817   21.329496
H    38.362764   32.401901   20.533455
H    38.206923   31.557204   18.381556
H    38.590993   29.837276   18.255397
H    37.246794   30.514397   17.354959
H    33.872446   25.158851   20.172589
H    35.035404   25.198238   21.512128
H    35.469227   24.477224   19.968642
H    37.469106   25.745889   20.304126
H    36.870452   25.606386   18.643146
H    37.607439   27.095152   19.205928
```

=== 5 (sextet) ===

```
h    39.049762   39.492161   22.461973
C    38.938615   39.532369   21.357725
H    37.911849   39.271008   21.093255
H    39.129940   40.560346   21.035065
S    40.143245   38.470380   20.482204
N    40.849091   35.917557   18.809127
C    41.233378   36.340982   17.557655
C    41.975456   35.421702   19.445343
C    42.650710   36.122149   17.397371
C    43.112051   35.578935   18.559240
C    41.923794   34.813250   20.697018
H    42.809919   34.291644   21.033778
C    40.845012   34.779660   21.577337
N    39.598453   35.322089   21.349461
C    38.895489   35.224986   22.531398
C    39.714036   34.552478   23.517641
C    40.908198   34.277561   22.930327
C    37.606736   35.713993   22.739903
H    37.187097   35.576806   23.731501
C    36.794510   36.363452   21.811218
C    35.433183   36.802115   22.029081
C    36.084055   37.267024   19.908936
N    37.168146   36.670764   20.518295
C    34.997678   37.348104   20.859550
C    36.043568   37.687640   18.578297
H    35.127742   38.177081   18.263444
C    37.049031   37.554576   17.618920
C    36.971109   37.980686   16.231373
N    38.301537   37.034903   17.869510
C    39.025220   37.152010   16.702092
C    40.381389   36.852501   16.584269
H    40.851320   37.042693   15.628369
Fe   39.102304   36.574167   19.757456
C    38.187323   37.725930   15.663304
h    38.538458   37.895017   14.723450
h    44.096406   35.388089   18.702260
h    36.050729   38.339702   15.761520
h    43.285154   36.569846   16.625180
h    41.778076   33.783420   23.367642
h    34.020002   37.784113   20.654593
h    34.865626   36.661725   22.955578
h    39.496180   34.532390   24.583193
O    37.037156   33.422435   19.329707
C    38.701646   26.320958   23.804115
C    37.549212   27.202443   23.409940
O    36.708612   27.643067   24.167355
O    37.585367   27.438894   22.085024
C    36.611022   28.339883   21.528122
C    35.365707   27.606483   21.090088
C    34.141648   27.917319   21.573256
C    33.852407   28.749868   22.791623
C    32.928280   27.395138   20.809081
O    31.753869   28.221195   21.073163
C    30.896171   27.839416   22.057945
O    31.043743   26.861318   22.755060
C    29.791250   28.898702   22.205447
O    30.320110   29.892636   23.097545
C    28.459861   28.361088   22.762486
N    28.551936   28.209301   24.212409
C    27.644557   27.403835   24.854290
O    26.959822   26.584749   24.245938
C    27.549083   27.558750   26.343618
C    28.473748   28.270823   27.118528
C    28.290719   28.386580   28.494873
C    27.190138   27.787891   29.107584
C    26.270523   27.069176   28.343132
C    26.452865   26.954567   26.968928
C    27.257832   29.237886   22.399954
C    26.118837   28.650843   21.836298
C    24.974406   29.410504   21.585707
C    24.950150   30.768061   21.906036
C    26.079646   31.363537   22.471942
C    27.223820   30.604929   22.719390
C    33.140285   27.422188   19.276743
C    34.598154   27.147623   18.789496
O    34.564391   26.119053   17.803515
C    35.315253   28.409164   18.148945
O    34.760977   28.635190   16.835236
C    35.497971   28.528961   15.681063
O    35.087636   29.112412   14.701840
C    36.793574   27.772357   15.624137
C    37.886575   28.480517   15.106414
C    39.111492   27.847326   14.936254
C    39.253244   26.492246   15.231433
C    38.157238   25.770510   15.703009
C    36.931697   26.406938   15.909962
C    35.278928   29.746134   18.988583
```

C	34.520976	30.948153	18.356466
O	33.093019	30.705576	18.458648
C	32.479807	31.005227	19.624532
C	30.990095	30.932549	19.477621
O	33.073368	31.340939	20.632841
C	34.824871	31.496612	16.943754
O	35.317770	32.748715	17.488142
C	34.879961	32.398057	18.819995
C	35.971443	32.654126	19.876656
C	36.508537	31.356685	20.497464
O	37.664987	31.648279	21.248747
C	36.702733	30.158765	19.489306
C	37.652919	30.481853	18.305792
C	37.392716	29.097911	20.401827
O	38.588749	28.940530	20.338933
C	35.470124	26.567873	19.956641
C	34.910057	25.192128	20.439198
C	36.908900	26.224727	19.494352
H	38.350101	25.609513	24.548923
H	39.124438	25.807663	22.940297
H	39.491200	26.929822	24.257290
H	36.363253	29.083465	22.290030
H	34.740871	28.888447	23.405385
H	33.412684	29.717422	22.526387
H	33.114100	28.232425	23.411235
H	32.665220	26.394365	21.146435
H	29.591208	29.326207	21.220004
H	30.608615	30.676201	22.591990
H	28.295433	27.360303	22.355166
H	28.906169	29.027355	24.688546
H	29.353222	28.716770	26.663624
H	29.003307	28.942776	29.094081
H	27.054132	27.871281	30.181194
H	25.418789	26.592577	28.821559
H	25.763237	26.394140	26.349914
H	26.121435	27.589291	21.610777
H	24.103800	28.939759	21.136113
H	24.061523	31.361627	21.710080
H	26.069535	32.422262	22.716650
H	28.096962	31.079213	23.157754
H	32.797078	28.382821	18.887506
H	32.499142	26.662318	18.816304
H	33.950905	26.404445	17.076556
H	36.355496	28.126931	18.000451
H	37.771132	29.526873	14.841296
H	39.950191	28.417752	14.560802
H	40.215759	26.012366	15.089185
H	38.253382	24.710896	15.918819
H	36.088692	25.853654	16.302128
H	34.693861	29.542460	19.879087
H	30.642577	31.696447	18.778227
H	30.682326	29.966506	19.075821
H	30.507101	31.100047	20.437713
H	33.918743	31.637327	16.348916
H	35.578098	31.008457	16.332078
H	33.989933	32.970732	19.080928
H	36.895750	33.433510	18.364978
H	35.547185	33.239559	20.697596
H	35.751525	31.023997	21.210782
H	38.212282	32.226726	20.685617
H	38.031635	31.502579	18.352740
H	38.509842	29.807533	18.302836
H	37.151688	30.372031	17.344756
H	33.841539	25.056417	20.270994
H	35.112670	25.067417	21.505186
H	35.417343	24.388956	19.908965
H	37.434783	25.694922	20.289654
H	36.839511	25.568557	18.625845
H	37.538642	27.065806	19.214938
